# Injury incidence and associated factors among soccer players during the 2021 African Cup of Nations competition

**DOI:** 10.12688/f1000research.148161.2

**Published:** 2025-01-13

**Authors:** Amr Chaabeni, Amine Kalai, Helmi Ben Saad, Yacine Zerguini, Montassar Tabben, Karim Chamari, Anis Jellad

**Affiliations:** 1 Physical Medicine and Rehabilitation, Faculty of Medicine University of Monastir, Monastir, Monastir, 5000, Tunisia; 2 Research Laboratory of Technology and Medical Imaging - LR12ES06, Center for Musculoskeletal Biomechanics Research, Faculty of Medicine, University of Monastir, Tunisia., Monastir, Tunisia; 3 University of Sousse, Farhat Hached Hospital, Research Laboratory LR12SP09 “Heart Failure”, Sousse, Tunisia; 4 Medical Committee, Confederation of African Football, 6th of October City, Giza Governorate, Egypt; 5 FIFA Medical Centre of Excellence Algiers, Alger, Algeria; 6 Aspetar, Orthopaedic and Sports Medicine Hospital, FIFA Medical Centre of Excellence, Doha, Qatar; 7 Naufar Wellness & Recovery Center, Doha, Qatar; 8 Higher Institute of Sport and Physical Education Ksar-Said, University of La Manouba,, Tunis, Tunisia

**Keywords:** Africa, Epidemiology, On-field injury, Athletes, Football, Sport

## Abstract

**Background:**

Understanding the epidemiology of soccer injuries during specific international competitions is essential for customizing preventive strategies. Several studies have reported outcomes related to international competitions but, to the best of our knowledge, there has been no investigation into the injury patterns during any African Cup of Nations (AFCON) tournaments. This study aimed to analyse the incidence and the characteristics of soccer injuries during the 2021 African Cup of Nations (AFCON), which took place in Cameroon from January 9 to February 6, 2022.

**Methods:**

A video-based analysis covering 52 matches was conducted by two independent consultant physicians. They reviewed injury replays to determine incidence and characteristics ( ***i.e.***; mechanisms, body location, match moment, player substitution, absence in the next match, and referee decisions).

**Results:**

The tournament involved 519 male players, comprising 275 African (ALP) and 244 Non-African (NALP) league players. Eighty-seven injuries occurred, with incidences of 1.7 and 48.8 injuries per match and per 1000 match hours, respectively. Injury incidence rose with competition stages, particularly contact mechanism injuries. Non-contact injuries (23/87) predominantly occurred after 60 minutes of play (19/23), with the thigh being the most frequently affected body part (18/87). Older age and playing time significantly correlated with injury occurrence (p=0.032 and p<0.001, respectively). NALP midfielders and forwards were notably injured by contact mechanisms (36/45) in the attacking zone. Although non-contact mechanisms were more common in ALP than NALP (13/42 vs. 9/45), the difference lacked statistical significance (p=0.240), with a higher rate of muscle injuries (13/42 vs. 10/45, p=0.001).

**Conclusion:**

Muscle injuries prevailed among professional soccer players in the 2021 AFCON, with older age and playing time identified as key associated factors. Muscle injuries were more prevalent in ALP compared to NALP.

## Introduction

Soccer is a universally popular sport, boasting over 265 million licensed players globally, of which 46 million reside in Africa. ^1^ According to the Federation International de Football Association (FIFA), there are 128983 soccer professional players worldwide, with Africa contributing 22525, as per the Professional Football 2019 Report. ^2^ The physical demands of soccer, characterized by repetitive and high-impact movements, put players at a high risk of injuries. ^3^ Epidemiological studies play a crucial role in sports medicine, both for enhancing athletic performance and for serving as the foundational first phase in developing and implementing effective injury prevention programs. ^4^^,^^5^ Importantly, injury characteristics can vary depending on geographical factors, such as climate and competition type. ^6^^,^^7^ For example, ACL injuries and ankle sprains are more prevalent in warmer climates, while achilles tendon injuries are more common in cooler climates. ^6^ The reason why region-specific data are necessary for more effective preventive measures.

Injury rates differ significantly across competitions. For instance, the rate is 32.3 injuries per 1000 hours of exposure in national leagues, whereas it rises to 41.1 in international tournaments. ^8^ Even within Africa, there is significant variability; the South African league reports 24.8 injuries per 1000 match hours, ^9^ in contrast to 113.4 in the Nigerian league. ^10^ The study of Zerguini et al. ^11^ investigating the EPFKIN league in Congo RDC revealed an alarming six injuries per game, equivalent to 182 injuries per 1000 hours of competition. Indeed, some regional competitions, such as the West Africa Football Union, exhibit even higher incidences up to 289 injuries per 1000 match hours. ^12^

Despite the abundance of injury data from various leagues and tournaments worldwide, there remains a significant gap in the literature concerning injury patterns specific to the African Cup of Nations (AFCON). AFCON is a key competition in both the global and African soccer contexts. It features both local and professional players, including those from major European leagues with special infrastructure. Additionally, Africa serves as a vital talent pool for soccer players. Understanding the epidemiology of injuries within this competition is essential not only for developing localized preventive strategies but also for enhancing player safety and performance across Africa.

Thus, this study aimed to fill this gap by analyzing the incidence and associated factors of injuries among soccer players during the 2021 AFCON. By focusing on this previously unexplored population, this research seeks to provide novel insights into injury trends, contributing to global sports medicine and informing future interventions tailored to African and international contexts.

## Methods

### Study design

This was a video-based descriptive observational study related to the 2021 AFCON, which took place in Cameroon from January 9 to February 6, 2022. The 2021 competition was postponed to 2022 because of the coronavirus disease 2019 pandemic. Twenty-four national team have participated in this competition ( Box 1) with 519 players ( ***i.e.***; 275 African (ALP) and 244 Non-African (NALP) league players). The 52 matches were kicked off at four different times: 14:00 (n=5), 16:00 (n=1), 17:00 (n=22), and 20:00-h (n=24) ( Box 2).

Box 1. **Teams who participated in the African cup of nations-2022 competition (n=24).**

Algeria; Burkina Faso; Cameroon; Cape Verde; Comoros; Ivory Coast; Egypt; Equatorial Guinea; Ethiopia; Gabon; Gambia; Ghana; Guinea; Guinea-Bissau; Malawi; Mali; Mauritania; Morocco; Nigeria; Senegal; Sierra Leone; South Africa; Sudan; Tunisia.

Box 2. **List and times (T) of the 52 matches played during the African cup of nations-2022 competition.**

**Table:** 

Round of groups: from 9 to 20 January					
Group A	Group B	Group C	Group D	Group E	Group F
Cameroon vs. Burkina Faso **(T**_**3**_ **)**	Senegal vs. Zimbabwe **(T**_**1**_ **)**	Ghana vs. Morocco **(T**_**3**_ **)**	Nigeria vs. Egypt **(T**_**3**_ **)**	Algeria vs. Sierra Leone **(T**_**1**_ **)**	Tunisia vs. Mali **(T**_**1**_ **)**
Cape Verde vs. Ethiopia **(T**_**4**_ **)**	Guinea vs. Malawi **(T**_**3**_ **)**	Comoros vs. Gabon **(T**_**4**_ **)**	Sudan vs. Guinea Bissau **(T**_**4**_ **)**	Equatorial Guinea vs. Ivory Coast **(T**_**4**_ **)**	Mauritania vs. Gambia **(T**_**3**_ **)**
Cameroon vs. Ethiopia **(T**_**3**_ **)**	Senegal vs. Guinea **(T**_**1**_ **)**	Morocco vs. Comoros **(T**_**3**_ **)**	Nigeria vs. Sudan **(T**_**3**_ **)**	Ivory Coast vs. Sierra Leone **(T**_**3**_ **)**	Gambia vs. Mali **(T**_**1**_ **)**
Cape Verde vs. Burkina Faso **(T**_**4**_ **)**	Malawi vs. Zimbabwe **(T**_**3**_ **)**	Gabon vs. Ghana **(T**_**4**_ **)**	Guinea Bissau vs. Egypt **(T**_**4**_ **)**	Algeria vs. Equatorial Guinea **(T**_**4**_ **)**	Tunisia vs. Mauritania **(T**_**3**_ **)**
Burkina Faso vs. Ethiopia **(T**_**3**_ **)**	Zimbabwe vs. Guinea **(T**_**3**_ **)**	Ghana vs. Comoros **(T**_**4**_ **)**	Egypt vs. Sudan **(T**_**4**_ **)**	Sierra Leone vs. Equatorial Guinea **(T**_**3**_ **)**	Mali vs. Mauritania **(T**_**4**_ **)**
Cape Verde vs. Cameroon **(T**_**3**_ **)**	Malawi vs. Senegal **(T**_**3**_ **)**	Gabon vs. Morocco **(T**_**4**_ **)**	Guinea Bissau vs. Nigeria **(T**_**4**_ **)**	Ivory Coast vs. Algeria **(T**_**3**_ **)**	Tunisia vs. Gambia **(T**_**4**_ **)**
**Round of 16: from 23 to 26 January **					
Burkina Faso vs. Gabon **(T**_**3**_ **)** ^*^					
Nigeria vs. Tunisia **(T**_**4**_ **)**					
Guinea vs. Gambia **(T**_**3**_ **)**					
Cameroon vs. Comoros **(T**_**4**_ **)**					
Senegal vs. Cape Verde **(T**_**3**_ **)**					
Morocco vs. Malawi **(T**_**4**_ **)**					
Ivory Coast vs. Egypt **(T**_**3**_ **)** ^*^					
Mali vs. Equatorial Guinea **(T**_**4**_ **)** ^*^					
**Quarter-final: from 29 to January 30**					
Gambia vs. Cameroon **(T**_**3**_ **)**					
Burkina Faso vs. Tunisia **(T**_**4**_ **)**					
Egypt vs. Morocco **(T**_**2**_ **)** ^*^					
Senegal vs. Equatorial Guinea **(T**_**4**_ **)**					
**Semi-final: February 2 and 3**					
Burkina Faso vs. Senegal **(T**_**4**_ **)**					
Cameroon vs. Egypt **(T**_**4**_ **)** ^*^					
**Match for 3** ^**rd**^ **place: February 5**					
Burkina Faso vs. Cameroon **(T**_**4**_ **)**					
**Final: February 6**					
Senegal vs. Egypt **(T**_**4**_ **)** ^*^					

**T**_**1**_: 14:00; **T**_**2**_: 16:00; **T**_**3**_: 17:00; **T**_**4**_: 20:00.

^*^ Match with extra-time

There was no need for an ethical committee approval as the data were taken from publicly available video footages. The study was conducted following the guidelines established by the STROBE statement. ^13^

### Study protocol

Two independent consultant physicians, ( ***AJ*** and ***AC*** in the authors’ list, with 17 and 4 years’ experience in sports medicine and physical rehabilitation, respectively), followed the live streaming of matches ( Box 2) and collected data related to every match. Potential biases, such as selection bias in injury reporting, were mitigated by comprehensive video replay review, capturing all visible match incidents. In case of discrepancy, the intervention of a third author (AK) was required.

An injury was defined as any event causing a match interruption of more than 15 seconds, during which a player appeared to be in pain or received medical attention, regardless of whether it resulted in absence from the match or training, consistent with previous studies ^14^^–^^16^

Injuries responding to the previous definition were included in the study. Injuries occurring during training or warm-up were excluded.

Each injury was characterized according to the characteristics of the:

i) Player’s age, playing position (goalkeeper, defender, midfielder, and forward), the league where the footballer was playing in at the moment of the competition ( ***i.e.***; NALP or ALP);
ii) Injury: Mechanism (contact vs. non-contact), body location (upper extremity, head and neck, trunk, thigh, knee, ankle), moment during the match, replacement after injury, absence next match, and eventual referee’s sanction;
iii) Match: Time, temperature and humidity (displayed at the presentation of each match just before the kick-off ), and competition stage ( ***e.g.***; round of groups, round of 16, quarterfinals, semi-finals, and finals (the third-place and final matches)).

Injury with contact mechanism was defined with any “physical contact with other player or object”, ^14^^,^^17^ otherwise it was considered as non-contact mechanism. Body location was classified by anatomical regions based on previous studies. ^8^^,^^18^

The moment of the injury was categorized into six “15-min” periods of standard match ^19^^,^^20^ ( ***i.e.***; 0-15, 16-30, 31-45, 46-60, 61-75, and 76-90 minutes), as the added extra-time was considered as a seventh period ( ***i.e.***; > 90 min).

### Statistical analysis

The Kolmogorov–Smirnov test was used to analyze the distribution of quantitative data. All quantitative data (except for time played) were normally distributed, and therefore were presented as means ± standard-deviation, and time played was expressed as median (interquartile). Mean difference (95% confidence interval) between two groups was calculated for the quantitative data. Categorical data were expressed as frequencies. For the analytical study, the Student's t-test was used to compare means for normally distributed variables, while the Mann-Whitney U test was employed to compare medians for non-normally distributed variables. The 2-sided Chi squared test was used to compare the categorical data and rates between groups.

The calculation of the incidence of injuries followed the recommendations of previous studies. ^15^^–^^17^ Injury frequencies are represented by the number of injuries per match played and by number of injuries per 1000 match hours. We have calculated the total hours of ‘match play’ as follows: 22 players × match duration using the factor 1.5, based on standard 90 min match play. ^15^^,^^16^ We decided to consider the 30 minutes possible extra-time during play offs ( ***i.e.***; rounds of 16, quarterfinal, semi-finals, and finals), we added the factor 0.5 for the matches requiring extra-time. Therefore, in this case we used the formula as follows: 22 players × match duration using factor 2.

The collected data were analyzed using a statistical software (StatSoft, Inc. (2014). STATISTICA (data analysis software system), version 12. www.statsoft.com, RRID: SCR_014213). The significance level was set at p<0.05.

## Results

During the 2021 AFCON competition, 52 matches were played, among which seven required the addition of extra-time ( Box 2). The total number of players who effectively participated in the AFCON was 519 (244 (47%) were NALP) ( Table 1). All NALP were European league players.

**Table 1.** African cup of nations (2022 competition): General characteristics.

Variable	Category	Value
Participating teams		24
Convoked players		672
Effectively participating players		519
Players’ league	Non-African	244 (47.0)
	African	275 (53.0)
Age of effectively participating players (Year)		26±3
Age of “non-African” league players (Year)		25±3
Age of African league players (Year)		26±3
Players’ positions (“non-African” league/African league)	Goalkeeper	41 (7/34)
	Defender	153 (76/77)
	Midfielder	177 (89/88)
	Forward	148 (72/76)
Total number of matches		52
Number of matches with extra time		7
Temperature (°C)		28±2
Humidity (%)		55±15
Time of match	14:00	5 (9.6)
	16:00	1 (1.9)
	17:00	22 (42.3)
	20:00	24 (46.1)

Quantitative data were mean ± standard deviation. Categorical data were number or percentage (%).

During the competition, 87 injuries were recorded, with 45 (51.7%) in NALP and 42 (48.2%) in ALP. These injuries occurred in 77 players, with 34 (44%) NALP and 43 (56%) ALP. The overall injury incidences were 1.7 and 48.8 injuries per match and per 1000 match hours, respectively. The average age of injured players was 26±4 years. The injury mechanisms were contact in 64 (74%) and non-contact in 23 (26%) cases. When analyzed by match time, 9 (10%) injuries occurred at 14:00, 43 (49%) at 16:00 and 17:00, and 35 (40.2%) at 20:00. Injury rates also varied by the round of the competition. A higher rate of injuries was recorded in playoff (2.5±1.5) compared to groups’ stage (1.3±1.2) (p=0.002).

**Figure 1.**
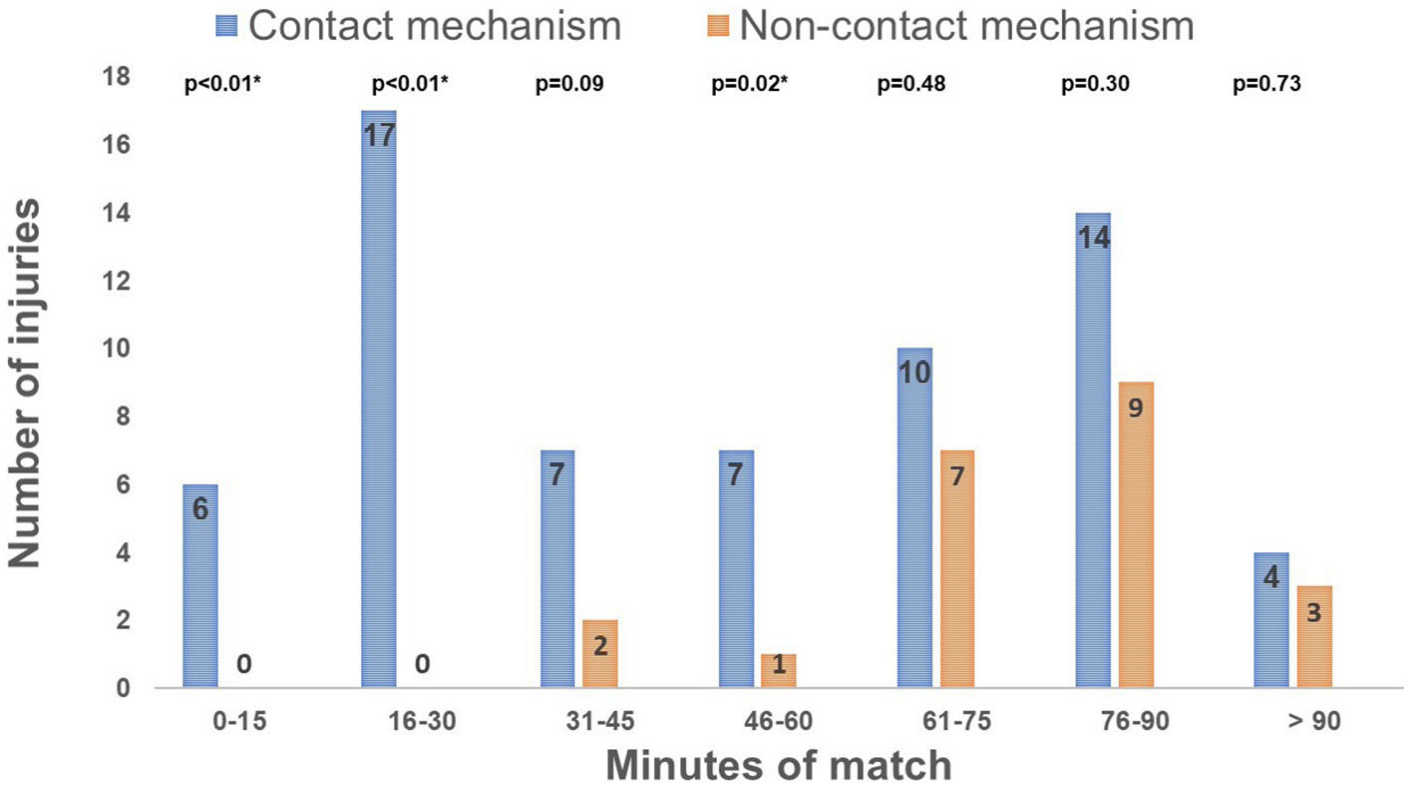
Distribution of injuries by mechanism and time intervals of the match. ^#^p-value (2-sided Chi squared test test) < 0.05: Comparison between the 2 groups.

Injuries resulting from contact mechanisms were mainly observed during the 16-30- and 76-90-minute intervals of the game (17/65 (26%) and 14/65 (21.5%) of injuries, respectively) ( Figure 1). Most non-contact injuries were observed after 60 minutes of play (19/23; 82.6%) ( Figure 1). The percentage of injuries due to contact mechanism compared to non-contact mechanism was significantly higher during the intervals of 0-15, 16-31 minutes, and 46-60 minutes ( Figure 1). Lower extremity injuries accounted for 65.5% of the total injuries (57/87). The most common injury locations were the thigh (20.7%), ankle (17.2%), and knee (16%).

**Figure 2.**
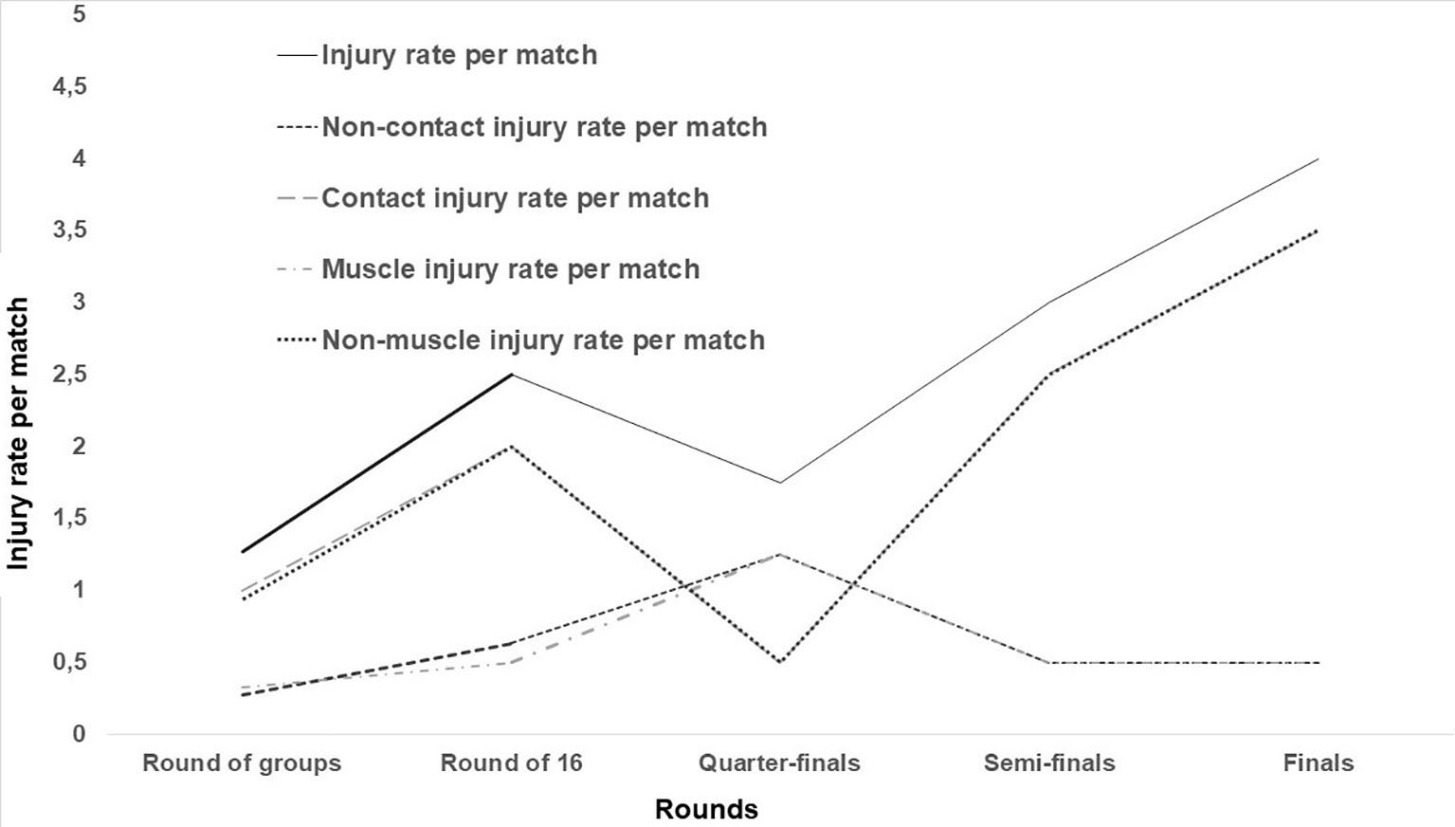
Injuries rate progression by match rounds.

The rate of injuries per match increased with the competition stage, especially those caused by contact mechanism ( Figure 2). The highest rate was noticed during the finals ( Figure 2).

Table 2 displays the factors associated with injuries. Compared to the group of non-injured players, the group of injured players was significantly older, had played longer than the non-injured ones, and included a higher percentage of goalkeepers.

**Table 2.** African cup of nations (2022 competition): Factors associated with injuries.

Factors	category	Injured players (n=77)	Non-injured players (n=442)	p-value
Age (Year)		27±4	26±3	0.032 ^*^
Player position	Goalkeeper	13 (17)	28 (6)	0.002 ^**#**^
	Defender	21 (27)	132 (30)	0.644 ^**#**^
	Midfielder	23 (30)	154 (35)	0.402 ^**#**^
	Forward	20 (26)	128 (30)	0.591 ^**#**^
Players’ league	«Non-African»	34 (44)	210 (48)	0.586 ^**#**^
	African	43 (56)	232 (52)	
Time played	Minutes	262 [171-425]	160 [73-270]	0.001 ^*^
Temperature (°C)		28.8±2.4	28.5±2.7	0.309 *
Humidity (%)		52.4±12.3	51.0±15.8	0.476 *

Quantitative data were mean±standard deviation, except for the time played which was median (interquartile). Categorical data were number (%).

^*^ p-value (Student t test or Mann-Witney U test) < 0.05: Comparison of quantitative data between the 2 groups.

^#^ p-value (2-sided Chi squared test test) < 0.05: Comparison of categorical data between the 2 groups.

Table 3 displays the comparison between NALP and ALP players in terms of injury characteristics. The injured NALP were significantly younger than the ALP. Non-contact mechanisms and muscle injuries were more common in the ALP compared to the NALP. In the NALP group, injuries were more frequent for midfielders and forwards than goalkeepers and defenders.

**Table 3.** Comparison between «non-African» league (NALP) and African league (ALP) players in terms of injury characteristics.

Data	Category	NALP (n=45)	ALP (n=42)	p-value
Age (Year)		25.9±4.0	27.9±3.6	0.017 ^*^
Age of players with muscle injuries (Year)		25±3.3	28.4±3.8	0.038 ^*^
Player position	Goalkeeper and defender	18 (40)	25 (60)	0.069
	Midfielder and forward	27 (60)	17 (40)	
Mechanisms of the injury	Non-contact	9 (20)	13 (31)	0.240
	Contact	36 (80)	29 (69)	
Type of injury	Muscle	10 (22.2)	13 (31)	0.001 ^*^
	Other	35 (77.8)	29 (69)	
Replacement of the player	Yes	15 (33.3)	23 (54.8)	0.044 ^*^
Absence next match	Yes	6 (13.3)	15 (35.7)	0.015 ^*^
Temperature (°C)		28.8±4.6	27.9±3.7	0.339
Humidity (%)		58.1±10.2	53.5±12.0	0.317

Quantitative data were mean±standard deviation. Categorical data were number (%).

^*^ p-value (Student t test or 2-sided Chi squared test) < 0.05: comparison between the 2 groups.

## Discussion

The present study identified injury rates of48.8 injury per 1000 match hours and 1.7 per match during the 2021 AFCON, and the rate of injuries per match increased with the competition stage. Injuries occurred mostly in goalkeepers. Non-contact injuries occurred mainly during the last third of the game. Injuries involved mainly lower extremity with the thigh being the most common concerned location. Injured players were significantly older and had longer amount of time played compared to those non-injured. NALP were injured mainly by contact mechanism, in the attacking zone of the field and injuries involved especially midfielders and forwards. Non-contact mechanism was more common in ALP with a higher rate of muscle injuries compared to NALP.

The documented incidence of injuries per 1000 match hours in the present study ( ***i.e.***; 48.8) is comparable to the 50.8 reported during the 2014 FIFA World Cup, ^17^ but is higher than those reported by Waldén et al. ^21^ during the 2004 European Football Championship (EURO) (36 per 1000 match hours) and by Bengtsson et al. ^22^ during the 2015/2016 and 2016/2017 Champions League seasons (20.3 per 1000 match hours) and 2016 Copa Libertadores (20.9 per 1000match hours). The documented incidence is lower than those reported during the FIFA World Cup hold in Korea/Japan 2002, Germany 2006, and South Africa 2010, respectively 81, 68.7, and 61.1 per 1000 match hours. ^15^^,^^16^^,^^23^ Moreover, the incidence of injuries per match we report ( ***i.e.***; 1.7 injuries) is comparable to the 1.68 reported during the 2014 FIFA World Cup ^17^ but ***(i)*** Lower than those noticed during FIFA World Cup (2.3 per match), Olympic Games (2.3 per match) and FIFA confederation cups (2.8 per match) from 1998 to 2012 ^24^; and ***(ii)*** Higher than those noticed during the 2017 Gold Cup ^14^ and EURO 2004, ^21^ 1.22 and 1.04 per match respectively. This discrepancy may potentially be due to the different type of competition/environment, the improvement in injuries prevention programs and the strict referees’ interventions. Indeed, the decrease in the injuries’ incidence in recent years was noticed by Junge et Dvořák ^17^ and explained by the referee sanctions’ evolution and the greater fair play by players. Differences in injury definitions and data collection methods across studies may account for variations in injury rates, highlighting the need for standardized reporting.

The increase of the rate of injuries with competition stages in our study is aligned with the conclusions of Yoon et al. ^25^ This can be explained by the cumulative fatigue during playoffs and the fact that physical commitment on the field is increasingly important as players approach the finals. Injuries during the later stages of the tournament may have influenced player availability and team outcomes, with key players missing subsequent matches. Discordantly, Junge et al. ^15^ reported no significant association between the incidence of injuries and the competition stages. The latter study investigated the 2002 FIFA World Cup, which included a higher number of matches and a different organization than continental competitions.

In accordance with our findings, the literature reported that, the incidence of injury within a match time increases especially after 60-min of the game. ^14^^,^^26^ As players spent more time on the field, the incidence of muscle injuries increased significantly. This is comparable to the results of Pangrazio and Forriol ^26^ and Chahla et al. ^14^ In fact, Pangrazio and Forriol ^26^ reported during the 2015 America cup that muscle strains happened in the last quarter of the match. Chahla et al. ^14^ reported an increase of injuries’ incidence with play time, especially between the 60 ^th^ and 75 ^th^ minute of play. Physical fatigue is the main explanatory factor according to some authors. ^14^^,^^27^ Other studies have reported the increase of injuries during the end of each half ^15^^,^^19^^,^^20^^,^^28^ with the high intensity of the match as possible explanation. ^15^ Non-contact injuries were most prevalent among midfielders and forwards, particularly during the last third of the match, likely due to accumulated fatigue. In contrasts with our results, Dvořák et al. ^23^ identified no differences in injuries’ distribution between the two halves.

Studies about soccer injuries identified that lower extremity is by far the most affected body part. Similarly to our findings, the thigh has previously been reported as the most commonly injured body part. ^8^^,^^17^^,^^19^^,^^26^^,^^29^ Soccer injuries epidemiology studies identified the older age as one of the main risk factors for muscle injuries. ^20^^,^^30^^–^^32^ In concordance with Pangrazio and Forriol ^26^ and Arnason et al., ^30^ we found that players who played for longer times were more exposed to injuries. Thus, the longer the exposure was (related to the long-time of practice), the higher the rate of injuries was. ^26^

There is no agreement in the literature about the rate of injuries by player position. We found that goalkeepers had the highest rate of injuries. Chahla et al. ^14^ and Arliani et al. ^33^ reported that forward players had the highest rate of injuries, while Chomiac et al. ^34^ reported the defenders as the most injured. This divergence in findings between studies may be explained by the difference of competitions type with the playing style potentially exposing specific player’s positions to a higher risk of being injured.

No previous study has investigated the injuries characteristics between players belonging to different continental leagues at the same competition. The higher rate of contact injuries noticed in the present study among NALP may be explained by the fact that these players are essentially forward and midfielder players with high level of skills, commonly injured in the attacking zone of the field. The fact that ALP in the present study had the highest rate of muscle injuries may be explained by the difference in training cultures between continents as it was reported between South American and Asian competitions and European competition. ^22^^,^^25^ While our study identified significant differences in injury patterns between African league players (ALP) and non-African league players (NALP), the underlying reasons for these variations were not fully explored. It is possible that geographical and cultural differences in training practices, such as variations in training intensity, recovery protocols, and match frequency, may contribute to these differences. Future research should focus on examining how these regional and cultural factors impact injury mechanisms and outcomes, as they likely play a role in the observed discrepancies between ALP and NALP players.

### Study limitations

The video-based analysis approach used in the present study did not include training and potential match’ warm-up-related injuries. This exclusion may underestimate the total injury burden, as training injuries are known to contribute significantly to overall injury rates. Furthermore, due to the study design, injury severity and recovery duration are not reported, missing an important piece of information of the recent epidemiological studies, ***i.e.*** injury burden. ^29^^,^^35^ Future studies should incorporate this information to better understand the long-term effects on player performance. Additionally, future research should address the limitations of this study by including training injuries, analyzing the impact of injury prevention measures, and evaluating the role of conditioning programs to determine their effectiveness in reducing injury rates, thereby providing a more comprehensive understanding of injury epidemiology.

## Conclusion

We report an injury incidence of 48.8 per 1000 match hours and a rate of 1.7 injuries per match during the 2021 AFCON. Older age and longer playing time were the main factors associated with injury. NALP players were mostly injured by contact mechanisms, while ALP players were mainly injured by non-contact mechanisms. ALP players had the highest rate of muscle injuries. These findings may highlight the importance of preventive interventions, especially targeting muscle injuries in ALP players.

## Ethics and consent

Ethical approval and written consent were not required.

## Data Availability

Zenodo: Excel data of the study titled: Injury incidence and associated factors among male soccer players during the 2021 African Cup of Nations competition: a pilot study. DOI: https://doi.org/10.5281/zenodo.10465671. ^36^ The project contains the following underlying data:
- Excel Data with description of injuries, matches and players’ characteristics. Data are available under the terms of the Creative Commons Attribution 4.0 International license (CC-BY 4.0). Zenodo: STROBE of the paper titled: Injury incidence and associated factors among soccer players during the 2021 African cup of nations’ competition. DOI: https://doi.org/10.5281/zenodo.10944493. ^37^
