# Observed events frequencies and associated factors among soccer players during the 2021 African Cup of Nations competition: A video-based exploratory analysis

**DOI:** 10.12688/f1000research.148161.3

**Published:** 2025-10-31

**Authors:** Amr Chaabeni, Amine Kalai, Helmi Ben Saad, Yacine Zerguini, Montassar Tabben, Karim Chamari, Anis Jellad

**Affiliations:** 1 Physical Medicine and Rehabilitation, Faculty of Medicine University of Monastir, Monastir, Monastir, 5000, Tunisia; 2 Research Laboratory of Technology and Medical Imaging - LR12ES06, Center for Musculoskeletal Biomechanics Research, Faculty of Medicine, University of Monastir, Tunisia., Monastir, Tunisia; 3 University of Sousse, Farhat Hached Hospital, Research Laboratory LR12SP09 “Heart Failure”, Sousse, Tunisia; 4 FIFA Medical Centre of Excellence Algiers, Alger, Algeria; 5 Medical Committee, Confederation of African Football, 6th of October City, Giza Governorate, Egypt; 6 Aspetar, Orthopaedic and Sports Medicine Hospital, FIFA Medical Centre of Excellence, Doha, Qatar; 7 Higher Institute of Sport and Physical Education Ksar-Said, University of La Manouba,, Tunis, Tunisia; 8 Naufar Wellness & Recovery Center, Doha, Qatar

**Keywords:** Africa, Epidemiology, On-field injury, Athletes, Football, Sport

## Abstract

**Background:**

Understanding the epidemiology of soccer injuries during specific international competitions is essential for customizing preventive strategies. Several studies have reported outcomes related to international competitions but, to the best of our knowledge, there has been no investigation into the injury patterns during any African Cup of Nations (AFCON) tournaments. This study aimed to analyze the observed events frequencies and their characteristics during the 2021 African Cup of Nations (AFCON), which took place in Cameroon from January 9 to February 6, 2022.

**Methods:**

A video-based analysis covering 52 matches was conducted by two independent consultant physicians. They reviewed “observed events” replays to determine frequencies and characteristics ( ***i.e.***; mechanisms, body location, match moment, player substitution, absence in the next match, and referee decisions).

**Results:**

The tournament involved 519 male players, comprising 275 African (ALP) and 244 Non-African (NALP) league players. Eighty-seven “observed events” occurred, with frequencies of 1.7 and 48.8 injuries per match and per 1000 match hours, respectively. Observed event frequency rose with competition stages, particularly contact mechanism injuries. Non-contact injuries (23/87) predominantly occurred after 60 minutes of play (19/23), with the thigh being the most frequently affected body part (18/87). Older age and playing time significantly correlated with observed event occurrence (p=0.032 and p<0.001, respectively). NALP midfielders and forwards were notably injured by contact mechanisms (36/45) in the attacking zone. Although non-contact mechanisms were more common in ALP than NALP (13/42 vs. 9/45), the difference lacked statistical significance (p=0.240), with a higher rate of muscle injuries (13/42 vs. 10/45, p=0.001).

**Conclusion:**

Muscle-related events were the most frequently observed during the 2021 AFCON, with older age and longer playing time identified as key associated factors. These events were more prevalent in ALP compared to NALP.

## Introduction

Soccer is a universally popular sport, boasting over 265 million licensed players globally, of which 46 million reside in Africa. ^1^ According to the Federation International de Football Association (FIFA), there are 128983 soccer professional players worldwide, with Africa contributing 22525, as per the Professional Football 2019 Report. ^2^ The physical demands of soccer, characterized by repetitive and high-impact movements, put players at a high risk of injuries. ^3^ Epidemiological studies play a crucial role in sports medicine, both for enhancing athletic performance and for serving as the foundational first phase in developing and implementing effective injury prevention programs. ^4^^,^^5^ Importantly, injury characteristics can vary depending on geographical factors, such as climate and competition type. ^6^^,^^7^ For example, ACL injuries and ankle sprains are more prevalent in warmer climates, while achilles tendon injuries are more common in cooler climates. ^6^ The reason why region-specific data are necessary for more effective preventive measures.

Injury rates differ significantly across competitions. For instance, the rate is 32.3 injuries per 1000 hours of exposure in national leagues, whereas it rises to 41.1 in international tournaments. ^8^ Even within Africa, there is significant variability; the South African league reports 24.8 injuries per 1000 match hours, ^9^ in contrast to 113.4 in the Nigerian league. ^10^ The study of Zerguini et al. ^11^ investigating the EPFKIN league in Congo RDC revealed an alarming six injuries per game, equivalent to 182 injuries per 1000 hours of competition. Indeed, some regional competitions, such as the West Africa Football Union, exhibit even higher incidences up to 289 injuries per 1000 match hours. ^12^

Despite the abundance of injury data from various leagues and tournaments worldwide, there remains a significant gap in the literature concerning injury patterns specific to the African Cup of Nations (AFCON). AFCON is a key competition in both the global and African soccer contexts. It features both local and professional players, including those from major European leagues with special infrastructure. Additionally, Africa serves as a vital talent pool for soccer players. Understanding the epidemiology of injuries within this competition is essential not only for developing localized preventive strategies but also for enhancing player safety and performance across Africa.

Thus, this study aimed to provide an exploratory, descriptive account of observed event-related stoppages during the 2021 AFCON, generating hypotheses that may inform future consensus-based surveillance research. By focusing on this previously unexplored population, this research seeks to provide novel insights into observed events trends, contributing to global sports medicine and informing future interventions tailored to African and international contexts.

## Methods

### Study design

This was a video-based descriptive observational study related to the 2021 AFCON, which took place in Cameroon from January 9 to February 6, 2022. The 2021 competition was postponed to 2022 because of the coronavirus disease 2019 pandemic. Twenty-four national team have participated in this competition ( Box 1) with 519 players ( ***i.e.***; 275 African (ALP) and 244 Non-African (NALP) league players). The 52 matches were kicked off at four different times: 14:00 (n=5), 16:00 (n=1), 17:00 (n=22), and 20:00-h (n=24) ( Box 2).

Box 1. **Teams who participated in the African cup of nations-2022 competition (n=24).**

Algeria; Burkina Faso; Cameroon; Cape Verde; Comoros; Ivory Coast; Egypt; Equatorial Guinea; Ethiopia; Gabon; Gambia; Ghana; Guinea; Guinea-Bissau; Malawi; Mali; Mauritania; Morocco; Nigeria; Senegal; Sierra Leone; South Africa; Sudan; Tunisia.

Box 2. **List and times (T) of the 52 matches played during the African cup of nations-2022 competition.**

**Table:** 

Round of groups: from 9 to 20 January					
Group A	Group B	Group C	Group D	Group E	Group F
Cameroon vs. Burkina Faso **(T**_**3**_ **)**	Senegal vs. Zimbabwe **(T**_**1**_ **)**	Ghana vs. Morocco **(T**_**3**_ **)**	Nigeria vs. Egypt **(T**_**3**_ **)**	Algeria vs. Sierra Leone **(T**_**1**_ **)**	Tunisia vs. Mali **(T**_**1**_ **)**
Cape Verde vs. Ethiopia **(T**_**4**_ **)**	Guinea vs. Malawi **(T**_**3**_ **)**	Comoros vs. Gabon **(T**_**4**_ **)**	Sudan vs. Guinea Bissau **(T**_**4**_ **)**	Equatorial Guinea vs. Ivory Coast **(T**_**4**_ **)**	Mauritania vs. Gambia **(T**_**3**_ **)**
Cameroon vs. Ethiopia **(T**_**3**_ **)**	Senegal vs. Guinea **(T**_**1**_ **)**	Morocco vs. Comoros **(T**_**3**_ **)**	Nigeria vs. Sudan **(T**_**3**_ **)**	Ivory Coast vs. Sierra Leone **(T**_**3**_ **)**	Gambia vs. Mali **(T**_**1**_ **)**
Cape Verde vs. Burkina Faso **(T**_**4**_ **)**	Malawi vs. Zimbabwe **(T**_**3**_ **)**	Gabon vs. Ghana **(T**_**4**_ **)**	Guinea Bissau vs. Egypt **(T**_**4**_ **)**	Algeria vs. Equatorial Guinea **(T**_**4**_ **)**	Tunisia vs. Mauritania **(T**_**3**_ **)**
Burkina Faso vs. Ethiopia **(T**_**3**_ **)**	Zimbabwe vs. Guinea **(T**_**3**_ **)**	Ghana vs. Comoros **(T**_**4**_ **)**	Egypt vs. Sudan **(T**_**4**_ **)**	Sierra Leone vs. Equatorial Guinea **(T**_**3**_ **)**	Mali vs. Mauritania **(T**_**4**_ **)**
Cape Verde vs. Cameroon **(T**_**3**_ **)**	Malawi vs. Senegal **(T**_**3**_ **)**	Gabon vs. Morocco **(T**_**4**_ **)**	Guinea Bissau vs. Nigeria **(T**_**4**_ **)**	Ivory Coast vs. Algeria **(T**_**3**_ **)**	Tunisia vs. Gambia **(T**_**4**_ **)**
**Round of 16: from 23 to 26 January **					
Burkina Faso vs. Gabon **(T**_**3**_ **)** ^*^					
Nigeria vs. Tunisia **(T**_**4**_ **)**					
Guinea vs. Gambia **(T**_**3**_ **)**					
Cameroon vs. Comoros **(T**_**4**_ **)**					
Senegal vs. Cape Verde **(T**_**3**_ **)**					
Morocco vs. Malawi **(T**_**4**_ **)**					
Ivory Coast vs. Egypt **(T**_**3**_ **)** ^*^					
Mali vs. Equatorial Guinea **(T**_**4**_ **)** ^*^					
**Quarter-final: from 29 to January 30**					
Gambia vs. Cameroon **(T**_**3**_ **)**					
Burkina Faso vs. Tunisia **(T**_**4**_ **)**					
Egypt vs. Morocco **(T**_**2**_ **)** ^*^					
Senegal vs. Equatorial Guinea **(T**_**4**_ **)**					
**Semi-final: February 2 and 3**					
Burkina Faso vs. Senegal **(T**_**4**_ **)**					
Cameroon vs. Egypt **(T**_**4**_ **)** ^*^					
**Match for 3** ^**rd**^ **place: February 5**					
Burkina Faso vs. Cameroon **(T**_**4**_ **)**					
**Final: February 6**					
Senegal vs. Egypt **(T**_**4**_ **)** ^*^					

**T**_**1**_: 14:00; **T**_**2**_: 16:00; **T**_**3**_: 17:00; **T**_**4**_: 20:00.

^*^ Match with extra-time

There was no need for an ethical committee approval as the data were taken from publicly available video footages. The study was conducted following the guidelines established by the STROBE statement. ^13^

### Study protocol

Two independent consultant physicians, ( ***AJ*** and ***AC*** in the authors’ list, with 17 and 4 years’ experience in sports medicine and physical rehabilitation, respectively), followed the live streaming of matches ( Box 2) and collected data and observed-events related to every match. Potential biases, such as selection bias in event reporting, were mitigated by comprehensive video replay review, capturing all visible match incidents. In case of discrepancy, the intervention of a third author (AK) was required. Given that our approach relied on video-coded interruptions rather than medical injury surveillance, incidents measures should be interpreted as observed events frequencies rather than true epidemiological incidence rates.

An event was defined as any observed incident causing a match interruption of more than 15 seconds, during which a player appeared to be in pain or received medical attention, regardless of whether it resulted in absence from the match or training, consistent with previous studies. ^14^^–^^16^ It is important to note that this approach represents a video-coded event analysis rather than a medical injury surveillance system, and therefore does not adhere strictly to international consensus injury definitions ( ***e.g.***; International Olympic Committee, Union of European Football Associations, FIFA). Events identified may reflect transient discomfort, tactical behavior, or precautionary stoppages, and should not be interpreted as confirmed medical diagnoses.

Observed events responding to the previous definition were included in the study. Events occurring during training or warm-up were excluded.

Each observed event was characterized according to the characteristics of the:

i) Player’s age, playing position (goalkeeper, defender, midfielder, and forward), the league where the footballer was playing in at the moment of the competition ( ***i.e.***; NALP or ALP);
ii) Incident: Mechanism (contact vs. non-contact), body location (upper extremity, head and neck, trunk, thigh, knee, ankle), moment during the match, replacement after injury, absence next match, and eventual referee’s sanction;
iii) Match: Time, temperature and humidity (displayed at the presentation of each match just before the kick-off ), and competition stage ( ***e.g.***; round of groups, round of 16, quarterfinals, semi-finals, and finals (the third-place and final matches)).

Observed event with contact mechanism was defined with any “physical contact with other player or object”, ^14^^,^^17^ otherwise it was considered as non-contact mechanism. Body location was classified by anatomical regions based on previous studies. ^8^^,^^18^

The moment of the observed event was categorized into six “15-min” periods of standard match ^19^^,^^20^ ( ***i.e.***; 0-15, 16-30, 31-45, 46-60, 61-75, and 76-90 minutes), as the added extra-time was considered as a seventh period ( ***i.e.***; > 90 min).

### Statistical analysis

The Kolmogorov–Smirnov test was used to analyze the distribution of quantitative data. All quantitative data (except for time played) were normally distributed, and therefore were presented as means ± standard-deviation, and time played was expressed as median (interquartile). Mean difference (95% confidence interval) between two groups was calculated for the quantitative data. Categorical data were expressed as frequencies. For the analytical study, the Student’s t-test was used to compare means for normally distributed variables, while the Mann-Whitney U test was employed to compare medians for non-normally distributed variables. The 2-sided Chi squared test was used to compare the categorical data and rates between groups.

Following previous studies, we calculated the number of observed events per match and per 1000 match hours. ^15^^–^^17^ We have calculated the total hours of ‘match play’ as follows: 22 players × match duration using the factor 1.5, based on standard 90 min match play. ^15^^,^^16^ We decided to consider the 30 minutes possible extra-time during play offs ( ***i.e.***; rounds of 16, quarterfinal, semi-finals, and finals), we added the factor 0.5 for the matches requiring extra-time. Therefore, in this case we used the formula as follows: 22 players × match duration using factor 2.

The collected data were analyzed using a statistical software (StatSoft, Inc. (2014). STATISTICA (data analysis software system), version 12. www.statsoft.com, RRID: SCR_014213). The significance level was set at p<0.05.

## Results

During the 2021 AFCON competition, 52 matches were played, among which seven required the addition of extra-time ( Box 2). The total number of players who effectively participated in the AFCON was 519 (244 (47%) were NALP) ( Table 1). All NALP were European league players.

**Table 1.** African cup of nations (2022 competition): General characteristics.

Variable	Category	Value
Participating teams		24
Convoked players		672
Effectively participating players		519
Players’ league	Non-African	244 (47.0)
	African	275 (53.0)
Age of effectively participating players (Year)		26±3
Age of “non-African” league players (Year)		25±3
Age of African league players (Year)		26±3
Players’ positions (“non-African” league/African league)	Goalkeeper	41 (7/34)
	Defender	153 (76/77)
	Midfielder	177 (89/88)
	Forward	148 (72/76)
Total number of matches		52
Number of matches with extra time		7
Temperature (°C)		28±2
Humidity (%)		55±15
Time of match	14:00	5 (9.6)
	16:00	1 (1.9)
	17:00	22 (42.3)
	20:00	24 (46.1)

Quantitative data were mean ± standard deviation. Categorical data were number or percentage (%).

During the competition, 87 events were recorded, with 45 (51.7%) in NALP and 42 (48.2%) in ALP. These events occurred in 77 players, with 34 (44%) NALP and 43 (56%) ALP. The overall observed event frequencies were 1.7 and 48.8 events per match and per 1000 match hours, respectively. These values represent stoppages associated with apparent discomfort, not medically confirmed injury rates. The average age of players with events was 26±4 years. The observed event mechanisms were contact in 64 (74%) and non-contact in 23 (26%) cases. When analyzed by match time, 9 (10%) events occurred at 14:00, 43 (49%) at 16:00 and 17:00, and 35 (40.2%) at 20:00. Observed events frequencies also varied by the round of the competition. A higher rate of events was recorded in playoff (2.5±1.5) compared to groups’ stage (1.3±1.2) (p=0.002).

**Figure 1.**
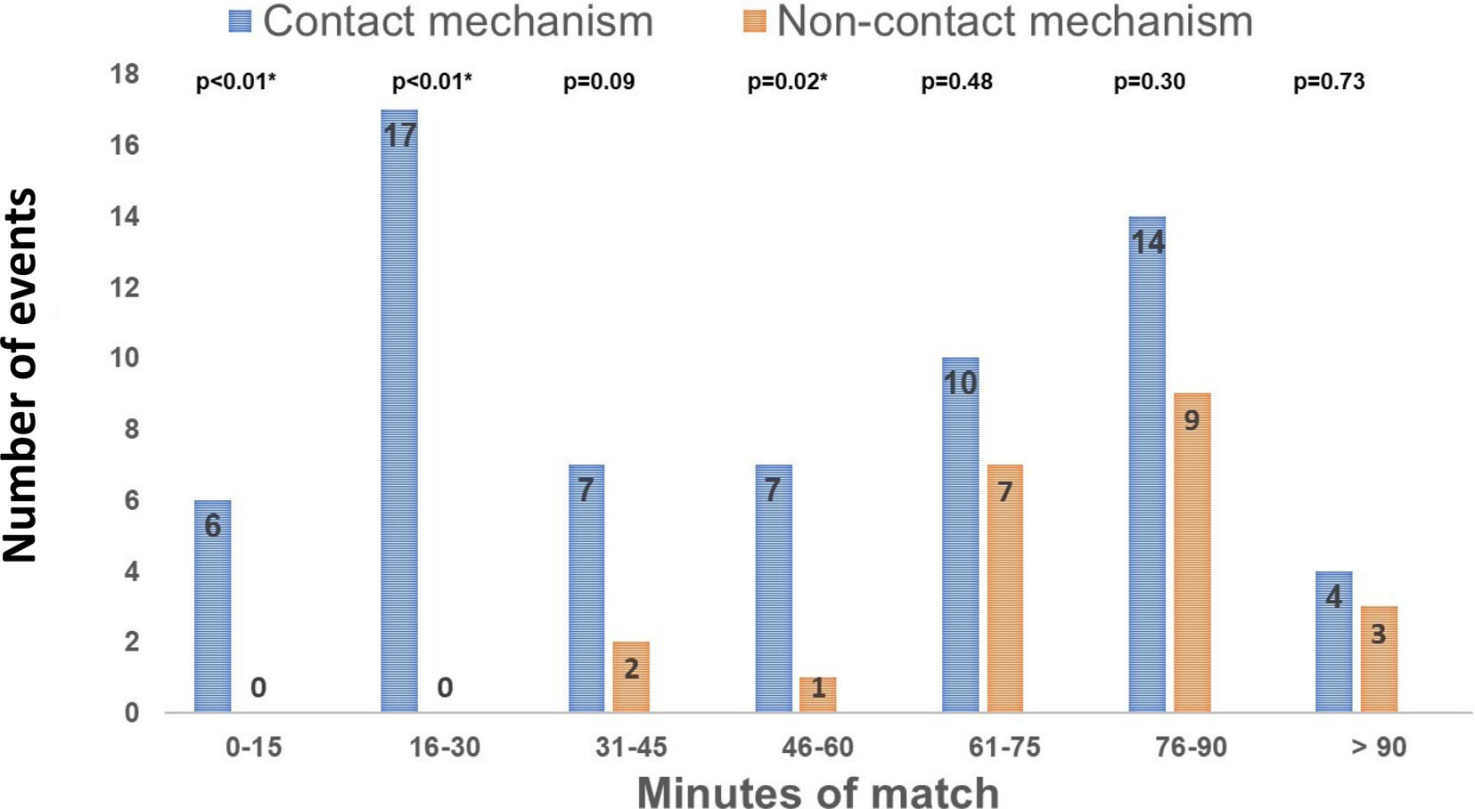
Distribution of observed events by mechanism and time intervals of the match. ^#^p-value (2-sided Chi squared test test) < 0.05: Comparison between the 2 groups.

Events resulting from contact mechanisms were mainly observed during the 16-30- and 76-90-minute intervals of the game (17/65 (26%) and 14/65 (21.5%) of observed events, respectively) ( Figure 1). Most non-contact events were observed after 60 minutes of play (19/23; 82.6%) ( Figure 1). The percentage of observed events due to contact mechanism compared to non-contact mechanism was significantly higher during the intervals of 0-15, 16-31 minutes, and 46-60 minutes ( Figure 1). Lower extremity events accounted for 65.5% of the total observed events (57/87). The most common observed events locations were the thigh (20.7%), ankle (17.2%), and knee (16%).

**Figure 2.**
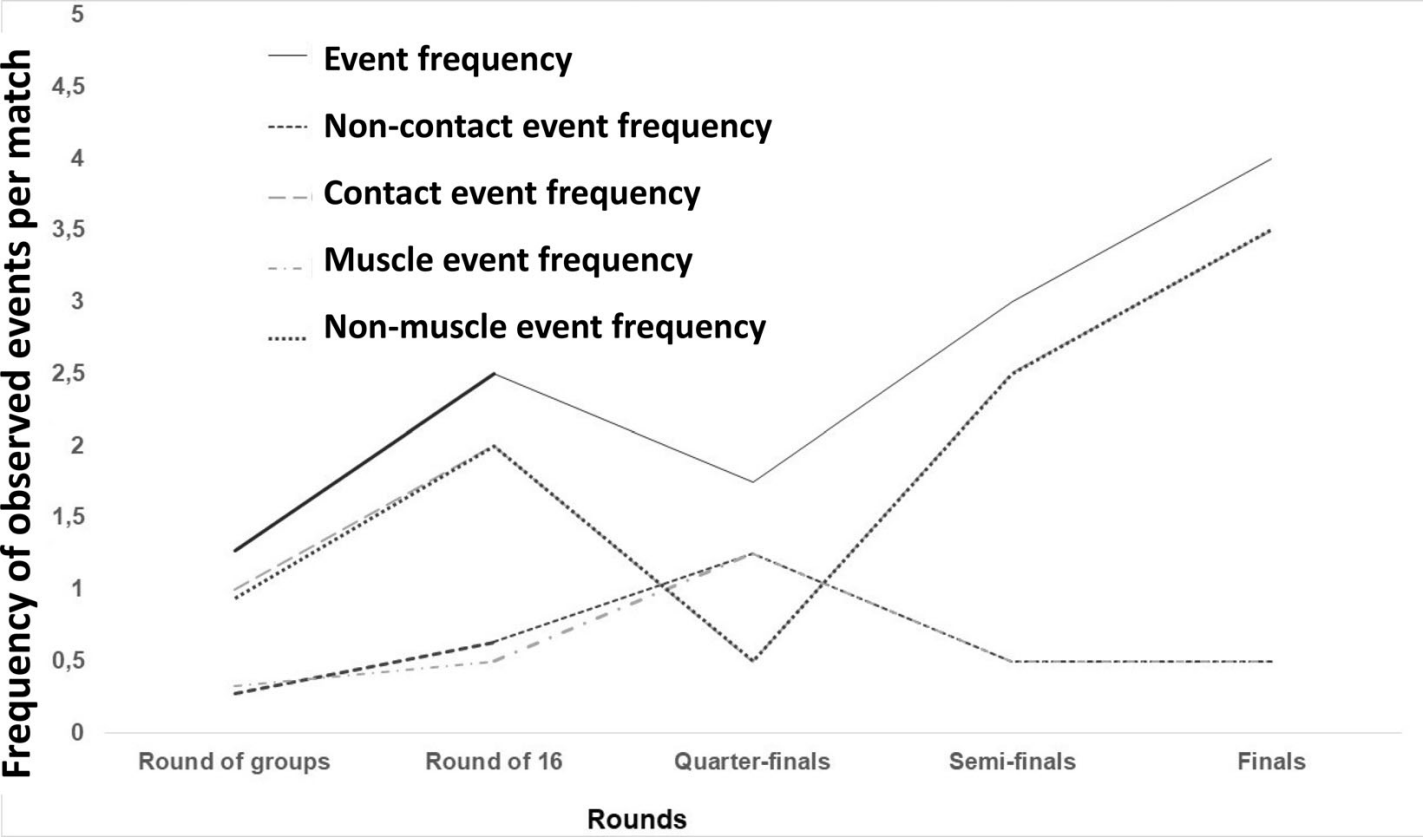
Progression of observed events by match rounds.

The frequency of observed events per match increased with the competition stage, especially those caused by contact mechanism ( Figure 2). The highest frequency was noticed during the finals ( Figure 2).

Table 2 displays the factors associated with observed events. Compared to the group of players free from events, the group of players with events was significantly older, had played longer than the players free from events, and included a higher percentage of goalkeepers.

**Table 2.** African cup of nations (2022 competition): Factors associated with observed events.

Factors	category	Players with events(n=77)	Players free from events(n=442)	p-value
Age (Year)		27±4	26±3	0.032 ^*^
Player position	Goalkeeper	13 (17)	28 (6)	0.002 ^**#**^
	Defender	21 (27)	132 (30)	0.644 ^**#**^
	Midfielder	23 (30)	154 (35)	0.402 ^**#**^
	Forward	20 (26)	128 (30)	0.591 ^**#**^
Players’ league	«Non-African»	34 (44)	210 (48)	0.586 ^**#**^
	African	43 (56)	232 (52)	
Time played	Minutes	262 [171-425]	160 [73-270]	0.001 ^*^
Temperature (°C)		28.8±2.4	28.5±2.7	0.309 *
Humidity (%)		52.4±12.3	51.0±15.8	0.476 *

Quantitative data were mean±standard deviation, except for the time played which was median (interquartile). Categorical data were number (%).

^*^ p-value (Student t test or Mann-Witney U test) < 0.05: Comparison of quantitative data between the 2 groups.

^#^ p-value (2-sided Chi squared test test) < 0.05: Comparison of categorical data between the 2 groups.

Table 3 displays the comparison between NALP and ALP in terms of observed events characteristics. The NALP with events were significantly younger than the ALP. Non-contact mechanisms and muscle observed events were more common in the ALP compared to the NALP. In the NALP group, observed events were more frequent for midfielders and forwards than goalkeepers and defenders.

**Table 3.** Comparison between «non-African» league players (NALP) and African league (ALP) players in terms of observed events characteristics.

Data	Category	NALP (n=45)	ALP (n=42)	p-value
Age (Year)		25.9±4.0	27.9±3.6	0.017 ^*^
Age of players with muscle observed events (Year)		25±3.3	28.4±3.8	0.038 ^*^
Player position	Goalkeeper and defender	18 (40)	25 (60)	0.069
	Midfielder and forward	27 (60)	17 (40)	
Mechanisms of the observed events	Non-contact	9 (20)	13 (31)	0.240
	Contact	36 (80)	29 (69)	
Type of observed events	Muscle	10 (22.2)	13 (31)	0.001 ^*^
	Other	35 (77.8)	29 (69)	
Replacement of the player	Yes	15 (33.3)	23 (54.8)	0.044 ^*^
Absence next match	Yes	6 (13.3)	15 (35.7)	0.015 ^*^
Temperature (°C)		28.8±4.6	27.9±3.7	0.339
Humidity (%)		58.1±10.2	53.5±12.0	0.317

Quantitative data were mean±standard deviation. Categorical data were number (%).

^*^ p-value (Student t test or 2-sided Chi squared test) < 0.05: comparison between the 2 groups.

## Discussion

The present study identified observed events’ frequencies of 48.8 per 1000 match hours and 1.7 per match during the 2021 AFCON. These figures represent descriptive match events rather than epidemiological injury frequencies, and therefore complement rather than replace consensus-based surveillance data. The rate of observed events per match increased with the competition stage. Observed events occurred mostly in goalkeepers. Non-contact events occurred mainly during the last third of the game. Observed events involved mainly lower extremity with the thigh being the most common concerned location. Players with events were significantly older and had longer amount of time played compared to those free from events. NALP were “injured” mainly by contact mechanism, in the attacking zone of the field and events involved especially midfielders and forwards. Non-contact mechanism was more common in ALP with a higher rate of muscle events compared to NALP.

The documented frequency of observed events per 1000 match hours in the present study ( ***i.e.***; 48.8) is comparable to the 50.8 injury incidence reported during the 2014 FIFA World Cup, ^17^ but is higher than incidences reported by Waldén et al. ^21^ during the 2004 European Football Championship (EURO) (36 per 1000 match hours) and by Bengtsson et al. ^22^ during the 2015/2016 and 2016/2017 Champions League seasons (20.3 per 1000 match hours) and 2016 Copa Libertadores (20.9 per 1000 match hours). The documented observed events frequency is lower than the injuries incidences reported during the FIFA World Cup hold in Korea/Japan 2002, Germany 2006, and South Africa 2010, respectively 81, 68.7, and 61.1 per 1000 match hours. ^15^^,^^16^^,^^23^ Moreover, the frequency of observed events per match we reported ( ***i.e.***; 1.7 observed events) is comparable to the 1.68 reported incidence of injuries during the 2014 FIFA World Cup ^17^ but ***(i)*** Lower than injuries incidences noticed during FIFA World Cup (2.3 per match), Olympic Games (2.3 per match) and FIFA confederation cups (2.8 per match) from 1998 to 2012 ^24^; and ***(ii)*** Higher than injuries incidences noticed during the 2017 Gold Cup ^14^ and EURO 2004, ^21^ 1.22 and 1.04 per match respectively. This discrepancy may potentially be due to the different type of competition/environment, the improvement in “injuries/events” prevention programs and the strict referees’ interventions. Indeed, the decrease in the injuries’ incidence in recent years was noticed by Junge et Dvořák ^17^ and explained by the referee sanctions’ evolution and the greater fair play by players. Differences in injury/events definitions and data collection methods across studies may account for variations in injury/events rates, highlighting the need for standardized reporting.

The increase of the rate of observed events with competition stages in our study is aligned with the conclusions of Yoon et al. ^25^ This can be explained by the cumulative fatigue during playoffs and the fact that physical commitment on the field is increasingly important as players approach the finals. Observed events during the later stages of the tournament may have influenced player availability and team outcomes, with key players missing subsequent matches. Discordantly, Junge et al. ^15^ reported no significant association between the incidence of injuries and the competition stages. The latter study investigated the 2002 FIFA World Cup, which included a higher number of matches and a different organization than continental competitions.

In accordance with our findings, the literature reported that, the incidence of injury within a match time increases especially after 60-min of the game. ^14^^,^^26^ As players spent more time on the field, the incidence of muscle injuries increased significantly. This is comparable to the results of Pangrazio and Forriol ^26^ and Chahla et al. ^14^ In fact, Pangrazio and Forriol ^26^ reported during the 2015 America cup that muscle strains happened in the last quarter of the match. Chahla et al. ^14^ reported an increase of injuries’ incidence with play time, especially between the 60 ^th^ and 75 ^th^ minute of play. Physical fatigue is the main explanatory factor according to some authors. ^14^^,^^27^ Other studies have reported the increase of injuries during the end of each half ^15^^,^^19^^,^^20^^,^^28^ with the high intensity of the match as possible explanation. ^15^ Non-contact observed events were most prevalent among midfielders and forwards, particularly during the last third of the match, likely due to accumulated fatigue. In contrasts with our results, Dvořák et al. ^23^ identified no differences in injuries’ distribution between the two halves.

Studies about soccer injuries identified that lower extremity is by far the most affected body part. Similarly to our findings, the thigh has previously been reported as the most commonly injured body part. ^8^^,^^17^^,^^19^^,^^26^^,^^29^ Soccer injuries epidemiology studies identified the older age as one of the main risk factors for muscle injuries. ^20^^,^^30^^–^^32^ In concordance with Pangrazio and Forriol ^26^ and Arnason et al., ^30^ we found that players who played for longer times were more exposed to events. Thus, the longer the exposure was (related to the long-time of practice), the higher the rate of observed events/injuries was. ^26^

There is no agreement in the literature about the rate of injuries/events by player position. We found that goalkeepers had the highest rate of observed events. Chahla et al. ^14^ and Arliani et al. ^33^ reported that forward players had the highest rate of injuries, while Chomiac et al. ^34^ reported the defenders as the most injured. This divergence in findings between studies may be explained by the difference of competitions type with the playing style potentially exposing specific player’s positions to a higher risk of being injured, and by differences in injury/events definitions.

No previous study has investigated the injuries/events characteristics between players belonging to different continental leagues at the same competition. The higher rate of contact events noticed in our study among NALP may be explained by the fact that these players are essentially forward and midfielder players with high level of skills, commonly “injured” in the attacking zone of the field. The fact that ALP in our study had the highest rate of muscle events may be explained by the difference in training cultures between continents as it was reported between South American and Asian competitions and European competition. ^22^^,^^25^ While our study identified significant differences in observed events between ALP and NALP, the underlying reasons for these variations were not fully explored. It is possible that geographical and cultural differences in training practices, such as variations in training intensity, recovery protocols, and match frequency, may contribute to these differences. Future research should focus on examining how these regional and cultural factors impact injury mechanisms and outcomes, as they likely play a role in the observed discrepancies between ALP and NALP.

### Study limitations

A key limitation is that our event coding did not follow consensus definitions of sports injury surveillance. Instead, we relied on video-based observations of match interruptions, which may overestimate or underestimate medically confirmed injuries. We therefore interpret these findings as descriptive indicators of match events rather than validated epidemiological injury data. The video-based analysis approach used in our study did not include training and potential match’ warm-up-related injuries. Furthermore, our design did not allow for collection of observed event severity, medical confirmation, or return-to-play outcomes. As a result, the burden and recovery impact of the observed events remain unknown, which limits comparability with consensus-based epidemiological studies. These omissions are related to feasibility constraints inherent to AFCON tournaments, where centralized medical surveillance data are not accessible. This exclusion may underestimate the total injury burden, as training injuries/events are known to contribute significantly to overall injury/events rates. Furthermore, due to the study design, observed events severity and recovery duration are not reported, missing an important piece of information of the recent epidemiological studies, ***i.e.*** injury/event burden. ^29^^,^^35^ Future studies should incorporate this information to better understand the long-term effects on player performance. Accordingly, our findings should be interpreted as exploratory, offering complementary descriptive insights into AFCON match events rather than definitive epidemiological estimates. Additionally, future research should address the limitations of this study by including training injuries/events, analyzing the impact of injury/event prevention measures, and evaluating the role of conditioning programs to determine their effectiveness in reducing injury/events rates, thereby providing a more comprehensive understanding of injury/events epidemiology.

## Conclusion

We report an observed events frequency of 48.8 per 1000 match hours and a frequency of 1.7 observed events per match during the 2021 AFCON. Older age and longer playing time were the main factors associated with observed events. NALPs were mostly ‘injured’ by contact mechanisms, while ALPs were mainly “injured” by non-contact mechanisms. ALPs had the highest rate of muscle observed events. These findings may highlight the importance of preventive interventions, especially targeting muscle incidents in ALPs.

## Ethics and consent

Ethical approval and written consent were not required.

## Data Availability

Zenodo: Excel data of the study titled: Observed events frequencies and associated factors among soccer players during the 2021 African Cup of Nations competition: A video-based exploratory analysis. DOI: https://doi.org/10.5281/zenodo.10465671. ^36^ The project contains the following underlying data:
- Excel Data with description of observed events, matches and players’ characteristics. Data are available under the terms of the Creative Commons Attribution 4.0 International license (CC-BY 4.0). Zenodo: STROBE of the paper titled: Observed events frequencies and associated factors among soccer players during the 2021 African Cup of Nations competition: A video-based exploratory analysis. DOI: https://doi.org/10.5281/zenodo.10944493. ^37^
