# Supplementary material for: Observed events frequencies and associated factors among soccer players during the 2021 African Cup of Nations competition: A video-based exploratory analysis
Source: F1000Res. 2025 Oct 31;13:392. Originally published 2024 Apr 25. [Version 3] doi: 10.12688/f1000research.148161.3 (PMC12770882; doi:10.12688/f1000research.148161.3)
Supplement: Peer review history

# Reviewer report: Reviewer response for version 3

**DOI:** 10.5256/f1000research.189910.r443373

**Published:** 2026-01-05

**Authors:** Mario Espada

**Affiliations:** 1 Instituto Politecnico de Setubal, Universidade de Lisboa, Life Quality Research Centre, Sport Physical Activity and Health Research & Innovation Center , University of Évora, Setúbal Municipality, Lisbon, Évora, Portugal

Dear Authors,

 I want to express my gratitude for the opportunity to review this manuscript.

 The document is well-organized and well-written on a global level.

 A major revision is recommended. Below are suggestions with section indication:

**Abstract:**

 Please consider the “p” in italics in the abstract and throughout the manuscript.

**Introduction:**

 Please make sure all abbreviations are in full in their first appearance in the manuscript. Please consider this suggestion throughout the manuscript.

 Please consider developing the introduction section to clearly present the study topic, research gap, and study aims.

**Methods:**

 Please describe all methodological details, for example, the equipment (manufacturer, version, city, and country), as well as the associated procedures in detail (including time of data collection, familiarization, human resources involved – academic background and experience), preferably with reference support.

 Statistical analysis – Please provide all details.

**Results:**

 Please revise the tables´ content and format, considering the journal template and instructions for authors.

 Please consider improving the quality of the figures.

 Please consider not placing tables 2 and 3 together to aid data interpretation by the readers.

**Discussion:**

 Please consider including more references in the discussion section to improve the quality os the text.

 In the end of the discussion section, please consider indicating suggestions for future research.

**Conclusions:**

 Please consider short and clear take-home messages, if possible, related to practical applications/implications.

 Please double-check the references format. For example, ref 36 title is not in bold.

 Please carefully revise the English details and document format, considering the journal template and instructions for authors.

Is the work clearly and accurately presented and does it cite the current literature?

Partly

If applicable, is the statistical analysis and its interpretation appropriate?

Partly

Are all the source data underlying the results available to ensure full reproducibility?

Yes

Is the study design appropriate and is the work technically sound?

Partly

Are the conclusions drawn adequately supported by the results?

Partly

Are sufficient details of methods and analysis provided to allow replication by others?

Yes

Reviewer Expertise:

Sports; Exercise; Physiology; Training; Testing.

I confirm that I have read this submission and believe that I have an appropriate level of expertise to confirm that it is of an acceptable scientific standard, however I have significant reservations, as outlined above.

# Reviewer report: Reviewer response for version 3

**DOI:** 10.5256/f1000research.189910.r429462

**Published:** 2025-12-27

**Authors:** Fearghal P Behan

**Affiliations:** 1 Trinity College Dublin, Dublin, Ireland

This study has clear methodological limitations, but data on injuries with the AFCON are necessary and this study may provide some novel (if limited) information from this tournament. The authors have attempted to address the valid concerns of previous reviewers and I won’t repeat these same clear limitations as the previous reviews are open and available.

 Abstract

 Older age and playing time correlated with event occurrence: from reading the paper I don’t think a correlation was undertaken on these variables. Perhaps rephrase.  

 NALP injuries: ’Notably’ injured by contact in the attacking zone, is this inferring that the notable element was the large percentage of contact injuries, or due to the location on the pitch? Is this notable as it is very different than the ALP?

 With a higher rate of muscle injuries in ALP than NALP? Might be worth writing this as a standalone sentence for clarity.

 Introduction:

 Last sentence in paragraph one reads awkwardly, perhaps something like: “Due to this climate-based injury differences, region-specific injury data may be beneficial for more effective injury prevention measures.”

 Methods

 The previous reviews have highlighted the severe limitations with the current methods. The validity or reliability of how this was conducted has still not been fully reinforced in the current version. The lack of ethical approval remains an obvious omission. No idea of intra- or inter-observer reliability of the current methods are clearly concerning. Especially since there appears to be a potential for subjectivity (i.e. a player appeared to be in pain) with deciding what is an ‘event’.

 Results

 Table 1. Why is non-African league in quotation marks? This is not the case elsewhere in the manuscript.  

 Figure 1. Font on x and y axis seem to be different.

 I am unsure how the examiners can tell the muscle is the source of injury from video analysis alone. Can this be elaborated upon within the methods please?

 I have some concerns regarding the repeated t-tests and Chi-squared tests. At a conservative count there are at least 18. The manuscript then interprets any values with a p below 0.05 as meaningful. The probability of a chance finding with so many uncorrected statistical tests should be acknowledged. Descriptive statistics appear more valid here, the use of inferential statistics seems to introduce a large potential for overinterpretation of incidental p values.

 Discussion

 The comparison to other incidence rates: is this comparing like with like? These papers used epidemiological data rather than the current methods. Presumably then these papers are not factoring in any incident which required on-field attention like the current analysis, making these difficult to compare. This applies throughout the discussion.

 Paragraph 5: ‘soccer injuries epidemiology studies’, this could be more concisely phrased.

 Paragraph 7: The claim that having a higher level of skill may explain their higher contact injuries seems questionable and not backed up by any data in the current study.

 A glaring omission throughout is the lack of acknowledgement of diving, time wasting etc. that would increase later in the game. The current methods do not control for this. If a player wished to stop play to reduce pressure on their team, and went down with a feigned injury, or exaggerating symptoms, the current analysis cannot factor this in. The low percentage of absences in the next match from the current observed events, may suggest that this may be part of the current numbers. This may explain more events later in the game, when game state jeopardy is higher. And the higher involvement of goalkeeper injuries, as the game cannot continue while they receive medical attention. This is not easy to quantify, or a savoury element of the sport, but needs to be confronted due to the current analysis not being driven by injury data and the prevalence of this behaviour in all high-level football tournaments.

Is the work clearly and accurately presented and does it cite the current literature?

Yes

If applicable, is the statistical analysis and its interpretation appropriate?

Partly

Are all the source data underlying the results available to ensure full reproducibility?

Yes

Is the study design appropriate and is the work technically sound?

Partly

Are the conclusions drawn adequately supported by the results?

Partly

Are sufficient details of methods and analysis provided to allow replication by others?

Partly

Reviewer Expertise:

Musculoskeletal health

I confirm that I have read this submission and believe that I have an appropriate level of expertise to confirm that it is of an acceptable scientific standard, however I have significant reservations, as outlined above.

# Reviewer report: Reviewer response for version 2

**DOI:** 10.5256/f1000research.176730.r409125

**Published:** 2025-09-11

**Authors:** Afxentios Kekelekis

**Affiliations:** 1 Aristotle University of Thessaloniki, Serres, Greece

The manuscript entitled “ *Injury incidence and associated factors among soccer players during the 2021 African Cup of Nations competition”* addresses an important competition and a population that has not been widely studied in the epidemiological literature. However, there are major methodological concerns that severely limit the validity of the findings.The principal issue lies in the exclusive reliance on video-based analysis to define and record injuries. The authors report that an injury was defined as any interruption in play lasting more than 15 seconds where a player appeared to be in pain or received medical attention. This definition does not align with internationally accepted consensus statements (IOC, FIFA, UEFA), which require medical confirmation and/or time-loss criteria. Because of this, it is impossible to determine whether the incidents recorded represented true musculoskeletal injuries, transient complaints such as cramps or knocks, or tactical simulations aimed at gaining advantage. Consequently, the calculated incidence rates (48.8 injuries/1000 match hours; 1.7 injuries/match) cannot be interpreted as true epidemiological measures but rather as counts of all stoppages associated with apparent discomfortFurthermore, no data were collected on training injuries, warm-up incidents, injury severity, diagnostic confirmation, or return-to-play duration.

 This omission substantially underestimates the overall burden of injury and prevents meaningful comparisons with existing epidemiological surveillance systems. The reliance on observational coding without validation or medical oversight introduces a high risk of misclassification and selection bias. Without these essential elements, the study should not be considered an epidemiological investigation, but rather a descriptive video analysis of match events. In its current form, the paper cannot provide robust conclusions about injury incidence, mechanisms, or risk factors. While the intention to describe injuries at AFCON is commendable, the methodological flaws undermine both internal validity and external generalisability. I would encourage the authors to explicitly acknowledge these limitations, refrain from presenting the results as epidemiological incidence rates, and instead frame the work as exploratory video-based observations. Only with medically confirmed data, standardised definitions, and comprehensive surveillance (including training and severity outcomes) can a study of this type contribute reliable information to the injury epidemiology literature.

Is the work clearly and accurately presented and does it cite the current literature?

No

If applicable, is the statistical analysis and its interpretation appropriate?

Partly

Are all the source data underlying the results available to ensure full reproducibility?

No

Is the study design appropriate and is the work technically sound?

No

Are the conclusions drawn adequately supported by the results?

No

Are sufficient details of methods and analysis provided to allow replication by others?

No

Reviewer Expertise:

Injury epidemiology, Injury risk analysis

I confirm that I have read this submission and believe that I have an appropriate level of expertise to state that I do not consider it to be of an acceptable scientific standard, for reasons outlined above.

## References

[1] : Consensus statement on injury definitions and data collection procedures in studies of football (soccer) injuries. *Scandinavian Journal of Medicine & Science in Sports* .2006;16(2) : 10.1111/j.1600-0838.2006.00528.x 83-92 10.1111/j.1600-0838.2006.00528.x 16533346

# Response

**Published:** 2025-10-17

**Authors:** Anis Jellad

Dear Reviewer,

 We sincerely thank you for your careful reading of our manuscript and for the constructive critiques, you have provided.

 Your observations have been invaluable in helping us clarify the scope, strengthen the methodology section, and better situate our findings within the literature.

 Below we provide a detailed, point-by-point response to the concerns raised.

**1. Concern: Use of video analysis and non-consensus injury definition**

**Response:** We agree with the reviewer that our injury definition does not follow the IOC/FIFA/UEFA consensus statements. To address this, we have clarified in the **Methods** section that our study is a video-coded event analysis rather than a medical injury surveillance study. We explicitly acknowledge in the **Discussion** section, that the coded events may include transient discomfort or tactical behavior and should not be interpreted as medically confirmed injuries. References to the consensus statements have been added to situate our approach within existing standards.

**2. Concern: Reported “incidence rates” are not true epidemiological measures**

**Response**: We appreciate this important point. To avoid misinterpretation, we have replaced the term “incidence rates” with “observed event frequencies” throughout the manuscript.

 In the **Title**, we changed “Injury incidence” by “observed event frequencies”.

 In the **Methods** section, we now specify that the reported numbers represent descriptive counts of stoppages associated with apparent discomfort rather than epidemiological injury rates.

 In the **Results** and **Discussion** sections, we emphasize that these values should be interpreted as descriptive indicators rather than epidemiological measures.

**3. Concern: Absence of training data, **warm-up incidents, injury **severity, **severity, diagnostic confirmation, **and return-to-play outcomes**

**Response**: We recognize the absence of training surveillance, injury severity grading, and return-to-play outcomes as important limitations. We have added explicit statements in the **Methods** and **Discussion** sections, noting that such data were not available within the scope of this study. In the **Limitations** subsection, we explaine that this precludes direct comparison with consensus-based epidemiological studies and that these omissions reflect feasibility constraints inherent to AFCON tournaments, where access to centralized medical surveillance data is limited.

**4. Concern: Framing of the study as epidemiology**

**Response**: We agree that our initial framing overstated the scope of the work. The manuscript has been revised to present the study as exploratory and hypothesis-generating. The title, abstract, and introduction section have been reframed to emphasize that our work provides descriptive insights into match events at AFCON, complementing rather than replacing consensus-based injury surveillance systems. The **Discussion** section explicitly acknowledges this positioning, clarifying that our findings are descriptive indicators and should not be considered definitive epidemiological estimates.

# Reviewer report: Reviewer response for version 2

**DOI:** 10.5256/f1000research.176730.r358773

**Published:** 2025-01-30

**Authors:** Pablo Prieto González

**Affiliations:** 1 Prince Sultan University, Riyadh, Riyadh Province, Saudi Arabia

I consider that the article is not index for the following reasons:

 The study demonstrates methodological weaknesses, as it relies solely on video-based analysis without incorporating direct player medical data or confirmed injury diagnoses. This limitation compromises the accuracy and reliability of the findings. Additionally, the research lacks approval from an Institutional Review Board (IRB), a critical requirement for studies involving human subjects or their data, even when using publicly available footage. The rationale for relying exclusively on video footage is insufficient, and the potential biases—such as missed injuries or misclassification of mechanisms—are not adequately addressed. While the focus on the African Cup of Nations (AFCON) offers novelty, the observational approach used undermines the generalizability of the findings to broader contexts or injury prevention strategies.

 I regret not being more positive on this occasion.

Is the work clearly and accurately presented and does it cite the current literature?

No

If applicable, is the statistical analysis and its interpretation appropriate?

Partly

Are all the source data underlying the results available to ensure full reproducibility?

Partly

Is the study design appropriate and is the work technically sound?

No

Are the conclusions drawn adequately supported by the results?

Partly

Are sufficient details of methods and analysis provided to allow replication by others?

No

Reviewer Expertise:

Sports training and injury prevention

I confirm that I have read this submission and believe that I have an appropriate level of expertise to state that I do not consider it to be of an acceptable scientific standard, for reasons outlined above.

# Reviewer report: Reviewer response for version 1

**DOI:** 10.5256/f1000research.162439.r300243

**Published:** 2024-09-13

**Authors:** Pablo Prieto González

**Affiliations:** 1 Prince Sultan University, Riyadh, Riyadh Province, Saudi Arabia

Thank you very much for giving me the opportunity to review this study. Below, I share my observations regarding the manuscript in each of its sections:

 INTRODUCCIÓN:

 The introduction of the article "Injury incidence and associated factors among soccer players during the 2021 African Cup of Nations competition" presents strengths and areas for improvement. Among the strengths, it provides relevant context, highlighting the popularity of soccer and the large number of registered players both in Africa and worldwide, establishing the importance of the study within the global soccer landscape. Additionally, the introduction underscores the relevance of epidemiological studies in sports medicine, both for enhancing athletic performance and for developing injury prevention programs. It also mentions that injury characteristics can vary according to geographical factors such as climate and competition type, reinforcing the need for specific data to implement effective preventive measures. A comparative analysis of injury rates in various competitions, both national and international, contextualizes the severity and variability of the issue. Finally, the introduction identifies a research gap: the lack of studies on injury characteristics in African Cup of Nations (AFCON) tournaments, justifying the study's objective.

 However, the introduction has several weaknesses. Some parts of the introduction could be clearer to improve understanding. Although geographical differences in injury incidence are mentioned, the study could developed deeper into how these differences influence the nature of injuries and the need for specific studies in different regions. It would be beneficial to provide more specific background on AFCON, highlighting its importance in the context of African and global soccer, to strengthen the study's justification. The transition to the study objectives could be smoother, improving the connection between identifying the research gap and the study's objectives for a more natural flow. There is room to improve clarity and particularly in depth in key aspects. Additionally, studying a previously unexamined population (players participating in the African Cup of Nations) does not constitute a novelty in itself unless it provides new insights into scientific knowledge.

 METHODS:

 Regarding the type of study, it is indicated as an analytical study. While this is not incorrect, it would be more accurate to specify that this is a video-based descriptive observational study. The STROBE-SIIS guidelines recommend providing a clear and detailed description of the study design, including the process for selecting injury events.

 Regarding the statement that "there was no need for an ethical committee approval," it is important to emphasize that ethical approval is necessary to protect the privacy and rights of the players, ensure informed consent, minimize potential harm, and comply with ethical regulations and guidelines. Review by an ethics committee provides an independent evaluation and ensures that the study is conducted in an ethical and responsible manner.

 Inclusion and Exclusion Criteria:

 Specific inclusion and exclusion criteria for injury events are not detailed. It is essential to specify these criteria to understand how it was determined which injuries were included in the analysis.

 Clearly Defined Outcomes:

 The definition of an injury incident is relatively general. STROBE-SIIS suggests clearly defining specific outcomes and justifying the criteria used. In this study, the definition of a "situation in which the match was interrupted" may be ambiguous.

 Sample Size Rationale:

 No justification is provided for the sample size or the formula used to calculate it. STROBE-SIIS recommends describing how the sample size was determined and providing adequate statistical justification for this.

 Variable Details:

 While some characteristics of the injuries and players are mentioned, a detailed description of all variables considered in the analysis is missing. STROBE-SIIS emphasizes the importance of accurately specifying and categorizing all variables. I consider that one of the main limitations of the study is the small number of variables studied, and the absence of a clear criterion to analyze in depth the mechanisms that cause injuries.

 Data Collection Methodology:

 The data collection methodology is based on observing live broadcasts and replays, but specific tools or methods used to ensure consistency and accuracy in data collection are not detailed. STROBE-SIIS suggests providing a detailed description of data collection methods, including any standardized tools or protocols used.

 Data Validation and Reliability:

 There is no mention of how the reliability of the data collected by the consulting physicians was validated. STROBE-SIIS recommends describing any validation processes or reliability tests applied to the data.

 Data Analysis and Statistical Considerations:

 Although some statistical analyses are described, there is a lack of justification for the choice of statistical tests used. STROBE-SIIS suggests providing a clear justification for the statistical techniques employed and explaining how potential confounders were addressed.

 Bias Information:

 Possible sources of bias and how they were addressed are not discussed. STROBE-SIIS recommends identifying and discussing potential sources of bias in the study and the strategies used to minimize them.

 Context and Confounding Data:

 Not enough information is provided on potential confounding factors, such as exact weather conditions or the previous physical condition of the players. STROBE-SIIS suggests considering and adjusting for potential confounders in the analysis.

 RESULTS

 The lack of clear definitions and criteria in the methodology section affects the results section. Although data on injuries are presented, there is no clear definition of the criteria used for injury categorization. While it specifies that injuries were either contact or non-contact and further breaks down the analysis by injury location and timing, the specific criteria for including injuries in the dataset are not detailed. This lack of clarity may hinder the evaluation of the validity and reliability of the findings.

 Inconsistent Data Presentation:

 Some data are presented as means with standard deviations while others are presented as medians with interquartile ranges, and the rationale for choosing these methods is not explained. For example, the choice of using the median for playing time should be justified, as the standard deviation is used for other continuous variables. The inconsistency in data presentation could affect the interpretability and comparability of the results.

 Absence of Statistical Power Analysis:

 The results section lacks information on statistical power analysis, which is essential to determine whether the sample size was adequate to detect significant differences. There is no mention of how the sample size was determined or justified, which could raise concerns about the validity of the statistical comparisons made.

 Potential Bias in Injury Reporting:

 The results indicate that injuries were recorded and analyzed, but there is no discussion of potential biases in injury reporting. For example, whether all injuries were uniformly reported and categorized, or if there was any observer bias in the injury assessment process, is not addressed.

 Limited Information on Contextual Factors:

 Factors such as weather conditions, listed as temperature and humidity, are mentioned but not thoroughly analyzed in relation to injury incidence. The impact of these variables should be examined more comprehensively to understand their effect on injury rates. Additionally, there is a lack of analysis regarding confounding factors such as player fatigue or previous injuries.

 Inadequate Analysis of Injury Mechanisms:

 While the results provide a general breakdown of injury mechanisms (contact vs. non-contact) and their occurrence by match time, there is a lack of detailed analysis of how different mechanisms relate to specific injury types or player positions. A more detailed examination could help in understanding injury patterns more comprehensively.

 Inadequate Analysis of Injury Impact:

 Although the results show variations in injury rates by match round and time, there is limited analysis of how these injuries affected player performance and match outcomes. The study does not explore how injuries impacted player availability or team performance, which could provide valuable insights into the broader implications of the findings.

 DISCUSSION AND CONCLUSION

 One of the main shortcomings is the lack of consideration for injuries occurring during training sessions, warm-ups, and cool-downs. This is a significant limitation, as injuries during these periods can substantially contribute to the overall injury burden. Excluding these injuries from the study may lead to an underestimation of the total incidence and severity of injuries, affecting the comprehensiveness of the findings.

 Additionally, the study does not report on the severity of the injuries or the duration of recovery. This omission is critical, as understanding the severity and recovery time of injuries provides crucial information on the injury burden and the long-term impact on players' health and performance.

 The study compares injury rates with several previous studies but does not delve deeply into the potential differences in study methodologies, such as variations in injury definitions, data collection methods, or competitive environments. These differences may introduce inconsistencies in comparative findings, making it difficult to draw definitive conclusions.

 The video-based analysis used in the study may introduce selection bias if certain injuries were less likely to be recorded or if injuries involving some players were not reported. Dependence on video footage may also overlook injuries that occurred off-camera or were inadequately captured, affecting the accuracy of reported injury rates.

 While the study reports injury rates by player position, it does not explore how different playing styles or positional demands might contribute to the observed injury patterns. Understanding these factors could provide a more nuanced view of injury risks associated with different player positions.

 The study highlights differences between players from different leagues (NALP vs. ALP) but does not thoroughly investigate how geographical or cultural differences in training practices might influence injury patterns. These differences could contribute to variations observed in injury mechanisms and types, but are not fully explored.

 The study also does not address whether players or teams had access to specific injury prevention measures, such as strength and conditioning programs or medical interventions. The presence or absence of such measures could influence injury rates and should be considered when interpreting the results.

 Despite presenting important limitations, the study does not thoroughly address the need for future research to address these shortcomings.

 As for the conclusion, it must be taken into account that the findings are specific to the 2021 AFCON competition, and may not be generalizable to other tournaments or leagues. Factors such as competition level, player conditioning, and environmental conditions may vary, impacting the applicability of the conclusions to different contexts.

Is the work clearly and accurately presented and does it cite the current literature?

No

If applicable, is the statistical analysis and its interpretation appropriate?

Partly

Are all the source data underlying the results available to ensure full reproducibility?

Partly

Is the study design appropriate and is the work technically sound?

No

Are the conclusions drawn adequately supported by the results?

Partly

Are sufficient details of methods and analysis provided to allow replication by others?

No

Reviewer Expertise:

Sports training and injury prevention

I confirm that I have read this submission and believe that I have an appropriate level of expertise to state that I do not consider it to be of an acceptable scientific standard, for reasons outlined above.

## References

[1] : International Olympic Committee consensus statement: methods for recording and reporting of epidemiological data on injury and illness in sport 2020 (including STROBE Extension for Sport Injury and Illness Surveillance (STROBE-SIIS)). *Br J Sports Med* .2020;54(7) : 10.1136/bjsports-2019-101969 372-389 10.1136/bjsports-2019-101969 32071062 PMC7146946

# Response

**Published:** 2025-01-07

**Authors:** Anis Jellad

Reviewer comments

 Response

**Comment**: Although geographical differences in injury incidence are mentioned, the study could developed deeper into how these differences influence the nature of injuries and the need for specific studies in different regions.

**Response:** As suggested, we added an example illustrating the influence of climate differences on injury nature.

**Comment**: It would be beneficial to provide more specific background on AFCON, highlighting its importance in the context of African and global soccer, to strengthen the study's justification.

**Response: **As suggested, we have added additional details about AFCON's significance, positioning it as a key tournament that features both local talent and international stars. Its importance for African football and global soccer development is emphasized, supporting the study's relevance.

**Comment**: The transition to the study objectives could be smoother, improving the connection between identifying the research gap and the study's objectives for a more natural flow. There is room to improve clarity and particularly in depth in key aspects. Additionally, studying a previously unexamined population (players participating in the African Cup of Nations) does not constitute a novelty in itself unless it provides new insights into scientific knowledge.

**Response: **We appreciate the reviewer's feedback on improving the flow and clarity. In response, we have refined the transition to better connect the identified research gap with the study’s objectives. We have also emphasized how this study contributes novel insights into injury epidemiology within the unique context of AFCON, offering valuable information for both regional and international injury prevention strategies.

**Comment**: Regarding the type of study, it is indicated as an analytical study. While this is not incorrect, it would be more accurate to specify that this is a video-based descriptive observational study.

**Response: **As suggested, the study design has been clearly redefined as a "video-based descriptive observational study" to align with reviewer recommendations and clarify the methodology.

**Comment**: The STROBE-SIIS guidelines recommend providing a clear and detailed description of the study design, including the process for selecting injury events.

**Response: **According to the STROBE-SIIS guidelines:

- “SIIS-4.1. Clearly specify which health problems are being observed”: this item is explained lines 122-125.
- SIIS-4.2. State explicitly which approach was used to record the health problem data, including all outcome measures or tools: this item is detailed lines 117-121.
- SIIS-4.3.State explicitly which coding system was used to classify the health problems:  lines 129-131
- SIIS-4.4. Where relevant, clearly describe how athletes were categorised: players were categorised based Player's age, playing position, and the league where the footballer was playing in at the moment of the competition (NALP or ALP); lines 127-128.

**Comment**: Regarding the statement that "there was no need for an ethical committee approval," it is important to emphasize that ethical approval is necessary to protect the privacy and rights of the players, ensure informed consent, minimize potential harm, and comply with ethical regulations and guidelines. Review by an ethics committee provides an independent evaluation and ensures that the study is conducted in an ethical and responsible manner.

**Response: **We stated that ethical approval was not required as the data were sourced from publicly available video footage. Additionally, the identities of the players were not disclosed (lines 114–115).

**Comment**: Inclusion and Exclusion Criteria:

 Specific inclusion and exclusion criteria for injury events are not detailed. It is essential to specify these criteria to understand how it was determined which injuries were included in the analysis.

**Response: **As suggested, we added an inclusion/exclusion criteria.

**Comment**: Clearly Defined Outcomes:

 The definition of an injury incident is relatively general. STROBE-SIIS suggests clearly defining specific outcomes and justifying the criteria used. In this study, the definition of a "situation in which the match was interrupted" may be ambiguous.

**Response: **As suggested, we reformulated the injury definition in order to raise ambiguity.

**Comment**: Sample Size Rationale:

 No justification is provided for the sample size or the formula used to calculate it. STROBE-SIIS recommends describing how the sample size was determined and providing adequate statistical justification for this.

**Response: **Sample size calculation was unnecessary, as all players participating in the tournament were included. In our case we conducted a population study.

**Comment**: Variable Details:

 While some characteristics of the injuries and players are mentioned, a detailed description of all variables considered in the analysis is missing. STROBE-SIIS emphasizes the importance of accurately specifying and categorizing all variables. I consider that one of the main limitations of the study is the small number of variables studied, and the absence of a clear criterion to analyze in depth the mechanisms that cause injuries.

**Response: **As suggested, we added a detailed description of missing variables. We reviewer for his valuable remark.

**Comment**: Data Collection Methodology:

 The data collection methodology is based on observing live broadcasts and replays, but specific tools or methods used to ensure consistency and accuracy in data collection are not detailed. STROBE-SIIS suggests providing a detailed description of data collection methods, including any standardized tools or protocols used.

**Response: **We thank the reviewer for their remark. Injuries were registered by the two authors (AJ and AC) independently and outcomes were compared at the end of each competition day.

**Comment**: Data Validation and Reliability:

 There is no mention of how the reliability of the data collected by the consulting physicians was validated. STROBE-SIIS recommends describing any validation processes or reliability tests applied to the data.

**Response: **Injuries were registered by the two authors (AJ and AC) independently and outcomes were compared at the end of each competition day. In case of discrepancy, the intervention of a third author (AK) was required.

 Details were added in the text (lines 121-122)

**Comment**: Data Analysis and Statistical Considerations:

 Although some statistical analyses are described, there is a lack of justification for the choice of statistical tests used. STROBE-SIIS suggests providing a clear justification for the statistical techniques employed and explaining how potential confounders were addressed.

**Response: **The authors thank the reviewer for their remark. For clarity, we have added a justification for the choice of statistical tests used.

**Comment**: Bias Information:

 Possible sources of bias and how they were addressed are not discussed. STROBE-SIIS recommends identifying and discussing potential sources of bias in the study and the strategies used to minimize them.

**Response: **A section has been added to address potential biases, such as selection bias in video-based injury analysis, and how these were mitigated.

**Comment**: Context and Confounding Data:

 Not enough information is provided on potential confounding factors, such as exact weather conditions or the previous physical condition of the players. STROBE-SIIS suggests considering and adjusting for potential confounders in the analysis.

**Response: **We acknowledge the lack details about potential confounding factors like player fatigue are now addressed.

**Comment**: Although data on injuries are presented, there is no clear definition of the criteria used for injury categorization. While it specifies that injuries were either contact or non-contact and further breaks down the analysis by injury location and timing, the specific criteria for including injuries in the dataset are not detailed. This lack of clarity may hinder the evaluation of the validity and reliability of the findings.

**Response: **We thank the reviewer for their valuable remark. This issue has been addressed in the Methods section under the inclusion criteria.

**Comment**: Inconsistent Data Presentation:

 Some data are presented as means with standard deviations while others are presented as medians with interquartile ranges, and the rationale for choosing these methods is not explained. For example, the choice of using the median for playing time should be justified, as the standard deviation is used for other continuous variables. The inconsistency in data presentation could affect the interpretability and comparability of the results.

**Response: **The mean and standard deviation are used when the distribution of data is approximately symmetrical. In contrast, the median and interquartile range are employed for skewed distributions, such as playing time, to better represent central tendency and variability. This approach ensures that the most appropriate statistical measures are used, enhancing the interpretability of the results.

**Comment**: Absence of Statistical Power Analysis:

 The results section lacks information on statistical power analysis, which is essential to determine whether the sample size was adequate to detect significant differences. There is no mention of how the sample size was determined or justified, which could raise concerns about the validity of the statistical comparisons made.

**Response: **We acknowledge the reviewer's comment regarding the absence of a statistical power analysis. Due to the nature of the study, which analyzed all available injury data from the 2021 AFCON, conducting a formal power analysis was not feasible. The study sample reflects the entire population of players and matches from the tournament, providing a comprehensive dataset. While this limits our ability to perform a prospective power calculation, we believe that analyzing the complete dataset ensures that the study captures all relevant injury patterns and associations.

**Comment**: Potential Bias in Injury Reporting:

 The results indicate that injuries were recorded and analyzed, but there is no discussion of potential biases in injury reporting. For example, whether all injuries were uniformly reported and categorized, or if there was any observer bias in the injury assessment process, is not addressed.

**Response: **A section has been added to address potential biases, such as selection bias in video-based injury analysis, and how these were mitigated.

**Comment**: Limited Information on Contextual Factors:

 Factors such as weather conditions, listed as temperature and humidity, are mentioned but not thoroughly analyzed in relation to injury incidence. The impact of these variables should be examined more comprehensively to understand their effect on injury rates. Additionally, there is a lack of analysis regarding confounding factors such as player fatigue or previous injuries.

**Response: **Weather conditions (temperature and humidity) have been analyzed, we haven’t find a relationship with injury incidence. We acknowledge the lack of analysis regarding confounding factors such as player fatigue or previous injuries

**Comment**: Inadequate Analysis of Injury Mechanisms:

 While the results provide a general breakdown of injury mechanisms (contact vs. non-contact) and their occurrence by match time, there is a lack of detailed analysis of how different mechanisms relate to specific injury types or player positions. A more detailed examination could help in understanding injury patterns more comprehensively.

**Response: **The analysis of injury mechanisms has been expanded to explore how different player positions and match contexts influence injury patterns.

**Comment**: Inadequate Analysis of Injury Impact:

 Although the results show variations in injury rates by match round and time, there is limited analysis of how these injuries affected player performance and match outcomes. The study does not explore how injuries impacted player availability or team performance, which could provide valuable insights into the broader implications of the findings.

**Response: **As suggested, we included a discussion on how injuries impacted player availability and potential performance in subsequent matches.

**Comment**: One of the main shortcomings is the lack of consideration for injuries ocurring during training sessions, warm-ups, and cool-downs. This is a significant limitation, as injuries during these periods can substantially contribute to the overall injury burden. Excluding these injuries from the study may lead to an underestimation of the total incidence and severity of injuries, affecting the comprehensiveness of the findings.

**Response: **We acknowledge this limitation and discuss how it may underestimate the total injury burden, suggesting it as an area for future research.

**Comment**: Additionally, the study does not report on the severity of the injuries or the duration of recovery. This omission is critical, as understanding the severity and recovery time of injuries provides crucial information on the injury burden and the long-term impact on players' health and performance.

**Response: **We address this limitation, indicating the need for future studies to incorporate data on injury severity and recovery time to better understand the overall burden.

**Comment**: The study compares injury rates with several previous studies but does not delve deeply into the potential differences in study methodologies, such as variations in injury definitions, data collection methods, or competitive environments. These differences may introduce inconsistencies in comparative findings, making it difficult to draw definitive conclusions.

**Response: **A discussion has been added to compare methodologies between this study and others, including how different definitions of injury and data collection techniques may influence findings.

**Comment**: The video-based analysis used in the study may introduce selection bias if certain injuries were less likely to be recorded or if injuries involving some players were not reported. Dependence on video footage may also overlook injuries that occurred off-camera or were inadequately captured, affecting the accuracy of reported injury rates.

**Response: **As suggested, some injuries may be not recorded, this discussion is added in limitation section

**Comment**: While the study reports injury rates by player position, it does not explore how different playing styles or positional demands might contribute to the observed injury patterns. Understanding these factors could provide a more nuanced view of injury risks associated with different player positions.

**Response: **As suggested, we provide a more detailed discussion of how different playing styles and positional demands may explain variations in injury patterns across player roles.

**Comment**: The study highlights differences between players from different leagues (NALP vs. ALP) but does not thoroughly investigate how geographical or cultural differences in training practices might influence injury patterns. These differences could contribute to variations observed in injury mechanisms and types, but are not fully explored.

**Response: **we acknowledge that differences in training loads, intensity, and preparation between African and non-African league players (ALP vs. NALP) could contribute to variations in injury mechanisms, this was beyond the scope of our current analysis. However, we have added a discussion in the manuscript, noting that future research should explore how regional training practices, competition schedules, and recovery protocols may impact injury risk and mechanisms, potentially contributing to the observed differences between ALP and NALP players.

**Comment**: The study also does not address whether players or teams had access to specific injury prevention measures, such as strength and conditioning programs or medical interventions. The presence or absence of such measures could influence injury rates and should be considered when interpreting the results.

**Response: **While detailed data on prevention measures were unavailable, we recommend that future studies investigate the influence of training and medical interventions on injury rates.

**Comment**: Despite presenting important limitations, the study does not thoroughly address the need for future research to address these shortcomings.

**Response: **A section on future research is included, emphasizing the need to study injury prevention strategies, the role of training, and to include training and warm-up injury data.

# Reviewer report: Reviewer response for version 1

**DOI:** 10.5256/f1000research.162439.r281561

**Published:** 2024-06-14

**Authors:** Fouad Chiha

**Affiliations:** 1 Universite Constantine 2, Constantine, Constantine Province, Algeria

Knowing the epidemiology of footballer injuries during international competitions is essential for developing preventive strategies. The study compared its results to those linked to other international competitions. This study aimed to characterize injuries contracted during the African Cup of Nations (AFCON) in Cameroon from January 9 to February 6, 2021.

 The study reports an injury incidence of 48.8 per 1000 match hours and a rate of 1.7 injuries per match. Muscle injuries were the highest and the main factors being advanced age and length of play.

 In conclusion, the study highlights the importance of preventive interventions. Addressing the use of preventive sessions (the FMS test is the best example) and recognized prophylactic means would have been relevant to put forward practical recommendations based on the results of the study. When discussing the results, we must pay attention to the characteristics of the competitions to be compared (climate, timing, match schedule, number of participating teams, number of matches played) to better appreciate the comparisons.

Is the work clearly and accurately presented and does it cite the current literature?

Yes

If applicable, is the statistical analysis and its interpretation appropriate?

Yes

Are all the source data underlying the results available to ensure full reproducibility?

Yes

Is the study design appropriate and is the work technically sound?

Yes

Are the conclusions drawn adequately supported by the results?

Yes

Are sufficient details of methods and analysis provided to allow replication by others?

Partly

Reviewer Expertise:

Training methodology –football- physical preparation- exercise physiology- monotoring..

I confirm that I have read this submission and believe that I have an appropriate level of expertise to confirm that it is of an acceptable scientific standard.
